# Supplementary material for: Transforming label-efficient decoding of healthcare wearables with self-supervised learning and “embedded” medical domain expertise
Source: Commun Eng. 2025 Jul 26;4:135. doi: 10.1038/s44172-025-00467-6 (PMC12297484; doi:10.1038/s44172-025-00467-6)
Supplement: Supplementary file 1 — Article File [file 44172_2025_467_MOESM1_ESM.pdf]

nature portfolio

Peer Review File

Transforming Label-Efficient Decoding of Healthcare Wearables with Self-Supervised Learning and "Embedded" Medical Domain Expertise

Corresponding Author: Dr Xiao Gu

This file contains all reviewer reports in order by version, followed by all author rebuttals in order by version.

Version 0:

Reviewer comments:

Reviewer #1

(Remarks to the Author)
Summary:

The authors propose a novel approach to extracting relevant information from wearable devices by combining traditional feature extractors with cutting-edge self-supervised deep learning frameworks. By integrating medical expertise into the deep learning process, their method demonstrates significant advantages across diverse datasets and methodologies. Specifically, leveraging a contrastive learning framework, which differentiates between positive samples (belonging to the same instance or class) and negative samples (from other instances or classes), the authors introduce a technique to identify additional positive samples within a domain-specific feature space. This knowledge is then transferred to a more general representation space. Furthermore, the inclusion of clustering mechanisms enhances the high-level learning of features. The authors validate the effectiveness of their method across various technologies, highlighting its robustness and adaptability.

Strengths:

1. The motivation behind the method and the overall study is promising, addressing a rapidly evolving field driven by the growing interest in wearable devices.
2. The proposed method is both novel and interesting. The concept of performing an additional search and integrating constraints derived from domain-specific features into a general self-supervised learning frameworks shows great promise for adaptation algorithms across various domains.
3. The authors provide a thorough analysis of their method across different types of data. The results demonstrate clear advantages of the proposed approach compared to existing self-supervised learning methods.
4. The authors thoughtfully discuss the limitations of their approach and outline potential future directions, including its real-time implementation on wearable devices.

Weaknesses:

1. Presentation of the Method:
- The abstract and introduction are overly vague, particularly the abstract. While the authors discuss their integration of domain expertise and self-supervised learning at a high level, they provide little to no detail about how this is achieved. This lack of specificity can be misleading and hinders the reader’s understanding of the method’s contributions. More methodological details should be incorporated into both the abstract and introduction.
- The method is divided into separate sections ("Overall Framework" and "Methods"), which creates confusion. These sections should be consolidated for coherence. The "Overall Framework" section, along with the figure, does not sufficiently explain the method. Key questions—such as how traditional ("old-school") features are combined with modern ("new-school") features, how domain-specific features are encoded, and how self-supervised learning (SSL) features are encoded—are only answered later in the "Methods" section. Including these details in the overview would improve clarity.
- The accompanying figure is difficult to interpret:
- The color coding (e.g., many purple features) is ambiguous, as not all features mapped to the embedding space are clearly labeled.

- The varying sizes of the features and the lack of clear column correspondence to inputs and representations add to the confusion.
- Subfigure (b) lacks context—how is it used in the framework? Are there sorting constraints, and how are "hard negatives" addressed? The corresponding section should be explicitly referenced.
- Visual clarity is an issue (e.g., white text on light gray background is hard to read). A legend is missing. The figure should be revised for better clarity and effectiveness.
- The "Methods" section is placed at the end, which disrupts the logical flow. It would be more helpful to present the method before the results.
- An important question: How is it ensured that domain-specific features and SSL features are combined? The guided aspect of the approach needs clearer explanation as it the main concept of the submission and the details are presented only in the end.

2. Data Annotation Costs:
The authors claim that annotating wearable device data is costly but provide no concrete estimates or references to support this assertion. Including such data, along with examples of annotations in the supplementary material, would be beneficial.

3. Claims About SSL Limitations:
The authors state that SSL methods fail to align with semantic knowledge required for downstream applications but do not provide references or analysis to support this claim. Adding supporting evidence is necessary.

4. Relevant Prior Work:
While the authors reference SSL methods and healthcare applications, the cited works (from 2021 and 2022) may not fully reflect the state-of-the-art. Are there more recent studies? Furthermore, the methods compared in Tables 2, 3, 5, and 6 are general SSL frameworks rather than domain-specific approaches for healthcare or wearable devices. Including comparisons to more relevant methods would strengthen the submission.

5. Connection to Applications:
A direct connection between the proposed approach and applications like “physical activity monitoring,” “wellness assessments,” and “fitness tracking” is lacking. How do experiments with ECG, EEG, and IMU translate into these applications? Although Table 1 lists tasks, it is neither referenced nor discussed. Are all tasks classification tasks? Clarification and integration into the narrative would improve the manuscript.

6. Analysis of Rare Signals:
The authors claim their approach significantly improves generalization for rare but important health signals but do not provide corresponding analysis or results. This claim needs to be supported with evidence or clearly pointed out in the results.

7. Implementation Details:
- It is unclear how waveforms are encoded. This information should be added.
- The effect of the number of neighbors chosen on performance is not addressed.
- The application of semi-supervised methods is ambiguous. Do the authors create pseudo-labels or merely add a supervised loss on top? This should be clarified in both the method and experimental sections.

8. Related Work Coverage:
The discussion of related work is missing. Recent advances in general SSL and representation learning for wearable devices, as well as semi-supervised frameworks (e.g., FixMatch), should be included as a separate section.

9. Ablations and Training Insights:
Additional ablation studies are needed:

- The influence of cluster-specific temperature parameters during training.
- The impact of the progressive application of prototypical contrastive learning (e.g., what happens if it is applied with a fixed weight of 1?).

10. BSCE Usage:
The authors mention using BSCE but do not explain its importance. Is it only used for downstream classification tasks? This needs clarification.

11. Unexpected Results in Table 2:
Linear probing with full supervision for IMU is significantly lower than for self-supervised training. Why does the "domain-guided" approach perform better here? What biases does it mitigate?

12. Redundant Figures:
The plots in Figure 4 appear redundant, as they add no new information to the manuscript. Consider removing them to streamline the presentation.

Minor Issues:

1. Repetitive Sentence in “Overall Framework” Section:
- The sentence *“…improve downstream validation performance, significantly improving downstream validation performance”* is redundant and needs to be revised for clarity and conciseness.
2. Word Choice in “Overall Framework” Section:
- Replace *“leveraged”* with *“leverage”* for grammatical consistency.
3. Augmentations:
- The authors follow prior works (e.g., 6, 11) on time series data, but the listed augmentations appear generic. Are there domain-specific augmentations tailored for this type of data that could be considered?
4. Section Naming:
- The title of the “Methods” section suggests a combination of existing approaches rather than presenting the proposed method. Consider renaming it to better reflect its content, such as “Proposed Methodology” or “Methodological Framework.”
5. Citations:
- Verify citation details for conferences such as ICLR and NeurIPS, as Google Scholar often lists incorrect publication years. For example, TS is published at ICLR 2023, not ICLR 2022.
6. Duplicate Tables:
- Tables 5 and 6 are identical and should be revised or removed to avoid redundancy.

Reviewer #2

(Remarks to the Author)
The authors claim to propose a novel approach to Self-Supervised Contrastive Learning in the domain of healthcare blending feature-guided selection of pairs (positive and negative) for downstream contrastive learning. They claim broad applicability to healthcare applications, including domains, devices and signals.

Strengths

Contrastive Learning may be a very useful and effective approach but requires lots of data. This problem is very severe in the health domain where data is scarce. Authors address the problem of generating data in this context using well-known techniques in unsupervised learning.
Applications to three different domains and biosignals are presented and evaluated.

Weakness
Healthcare: authors claim a Unified framework for varied types of healthcare wearables. They should down-scale their claims since they work on four datasets (EEG, ECG and Activity Recognition) which are not necessarily representative of healthcare (sub) domains and applications within.
The adjective “semantic” is mentioned 33 times. Given its role in the concept formulations and result presentations, a definition is required. For instance (from the introduction) improved search for “local semantic relationship” is claimed as a novel contribution. In the rest of the paper they mention “intricate semantic relationships” as well as “local” and “global” semantic relationships. Last but not least, why “..Data augmentation techniques, including temporal jittering, scaling, magnitude warping, Gaussian noise, and cutout, were applied to generate diverse views of the data following previous works” would not affect the “semantic relationship” of features ?. This is a problematic aspect in the comprehension of the paper and its parts.
Claims that are exaggerated and would require appropriate in-depth analysis of the cited paper. For instance “..deep learning have substantially improved the automatic extraction of semantic information from raw time series data (Ravi, D. et al. 2016)” : the mentioned which is relatively old , does not cover the semantic information of raw signals as processed by DL models.This is a problematic aspect in the comprehension of the paper and its parts.
The word “standard” or “established”are mentioned 4 times, each, in key contexts without expanding what is meant or given an example or a reference. For instance: “..across all datasets, standard processing was applied.” or “Domain-knowledge-informed features were extracted from each modality using established toolboxes and following clinical guidelines.“ This is a problematic aspect in the comprehension and replicability of the paper experiments. Experimental material and code should include all the necessary information for replicability.
“Domain Expertise Informed Features”: the reported features are domain agnostics and they may be relevant for a particular (sub)domain and application only if a meaning is formulated and associated.
Overall Self-Supervised Learning Performance : for fairness comparisons with latest deep representation learning models should be included in the experimental plan. Also authors should comment on the types of models reported in Table 2 and whether those results are replicated from original papers or else.
t-SNE plots in Figure 4 are not useful for contrasting the two approaches.

Editorial:
-Fig.1: Graphics are not clear. Specifically in the c) part, middle part and rightmost part the relation of color, edges, features is messy.

-”we conducted experiments across three types of wearable sensors: electrocardiogram (ECG), electroencephalogram (EEG), and inertial measurement unit (IMU).” . .ECG etc. are signals and not sensors.

Version 1:

Reviewer comments:

Reviewer #1

(Remarks to the Author)
I thank the authors for their thorough response. I have carefully reviewed their replies to my concerns and find them convincing; they significantly improve the quality of the submission.

One remaining concern:

Figure 2 has improved, but there are still some unclear elements. In the lower part of the figure, different features appear with either filled or unfilled backgrounds and in various colors (purple, black, gray). The meaning of these visual distinctions is not clear— a legend is needed. Additionally, there is an arrow running from top to bottom in the center, but it is not evident how the top and bottom sections are connected, either through color coding or any other means. The connection on the right side is clearer in comparison.

Open Access This Peer Review File is licensed under a Creative Commons Attribution 4.0 International License, which permits use, sharing, adaptation, distribution and reproduction in any medium or format, as long as you give appropriate credit to the original author(s) and the source, provide a link to the Creative Commons license, and indicate if changes were made.
In cases where reviewers are anonymous, credit should be given to 'Anonymous Referee' and the source.
The images or other third party material in this Peer Review File are included in the article's Creative Commons license, unless indicated otherwise in a credit line to the material. If material is not included in the article's Creative Commons license and your intended use is not permitted by statutory regulation or exceeds the permitted use, you will need to obtain permission directly from the copyright holder.
To view a copy of this license, visit https://creativecommons.org/licenses/by/4.0/

Response to review comments

COMMSENG-24-0634

We thank the reviewers for the thorough reading our manuscript and providing constructive feedback to further clarify our work. Extensive modifications have been made to the paper based on the comments received and we have attached herewith a point-by-point explanation of all the changes made to the manuscript. The text highlighted in blue corresponds to the revised text or figure/table number in the paper.

In summary, the major changes made to the paper include:

- **Improved Methodological Clarity:** Revised and reorganized relevant sections to clearly explain how domain knowledge is integrated, supported by revised figures. (C 1.1, C 1.2, C 1.4, C 1.5, C 1.13)
- **Conceptual and Terminology Clarifications:** Defined "semantic relationships" in wearable signals, clarified their robustness under augmentations, and refined ambiguous terms like "standard" and "established" with concrete examples. (C 2.2, C 2.3, C 2.4)
- **Enhanced Visuals:** Revised Figure 1 for clarity and updated t-SNE visualizations to better illustrate inter-subject generalization. (C 1.1, C 1.3, C 1.17, C 2.7, C 2.8)
- **Extended Comparison and Modalities:** Added comparisons to recent SSL and foundation models (e.g., MOMENT, Chronos, PaPaGei), and included PPG as a fourth modality to demonstrate generalizability. (C 1.8, C 1.9, C 2.1, C 2.6)
- **Additional Experiments:** Added analysis on neighbor number, prototype loss scheduling, and rare-class performance. Clarified augmentation robustness. (C 1.11, C 1.12, C 1.14, C 2.5)
- **Implementation Clarifications:** Detailed waveform encoding, semi-supervised setup (no pseudo-labels), use of BSCE, and dataset/implementation details. (C 1.6, C 1.12, C 1.15, C 1.16, C 1.20, C 2.9)
- **Extended Related Work and Context:** Expanded literature coverage in Supplement, and discussed real-world labeling challenges for wearables. (C 1.6, C 1.13, C 2.6)

All minor comments and typographical errors (C 1.18, C 1.19,C 1.21, C 1.22, C 1.23, C 2.9) have been addressed and rectified in the revised manuscript.

In the following we address the reviewers' comments point by point.

Reviewer 1

C 1.1 — The abstract and introduction are overly vague, particularly the abstract. While the authors discuss their integration of domain expertise and self-supervised learning at a high level, they provide little to no detail about how this is achieved. This lack of specificity can be misleading and hinders the reader's understanding of the method's contributions. More methodological details should be incorporated into both the abstract and introduction.

Reply: Many thanks for pointing out this issue. We have substantially revised these sections to improve the delivery of our methodology and its associated contributions, via the following two aspects.

(1) rephrasing the abstract and introduction part.

We have substantially revised both sections to clearly articulate how domain expertise is integrated into our self-supervised learning framework. In particular, we provide a concise yet specific description of the key methodological components, including how domain-informed features are used to guide contrastive pair selection and enhance semantic alignment in representation learning.

Below are relevant revisions from the main paper.

"(Page 1 of main paper) **Abstract**: Healthcare wearables are transforming health monitoring, generating vast and complex data in everyday free-living environments. While supervised deep learning has enabled significant advances in interpreting such data, it remains heavily dependent on large labeled datasets, which are often difficult and expensive to obtain in clinical practice. Self-supervised contrastive learning (SSCL) provides a promising alternative by learning from unlabeled data, but conventional SSCL frequently overlook important physiological similarities by treating all non-identical instances as unrelated, which can result in suboptimal representations. In this study, we revisit the enduring value of domain knowledge "embedded" in traditional domain feature engineering pipelines and demonstrate how it can be used to guide SSCL. We introduce a novel framework that integrates clinically meaningful features—such as heart rate variability from electrocardiograms (ECGs)—into the contrastive learning process. These features guide the formation of more relevant positive pairs through nearest-neighbour matching, and promote global structure through clustering-based prototype representations. Evaluated across diverse wearable technologies, our method achieves comparable performance with only 10% labeled data, compared to conventional SSCL approaches with full annotations for fine-tuning. This work highlights the indispensable and sustainable role of domain expertise in advancing machine learning for real-world healthcare, especially for healthcare wearables."

"(Page 2 of main paper) **Introduction:** Building on this insight, we propose a domain-knowledge-guided self-supervised contrastive learning (SSCL) framework that incorporates and leverages *"old-school"* domain-knowledge-informed features to guide *"new-school"* SSCL. Our framework extends the standard instance-level contrastive learning paradigm (as shown in Figure 9), in which two augmented views of the same signal are treated as positive pairs and all other signals in the batch are treated as negatives. This conventional setup, however, does not account for the semantic similarities between distinct patients or repeated measurements—potentially treating clinically similar signals as dissimilar during training. To address this, we incorporate domain-specific signal features to guide the formation of more meaningful pairings, thereby improving representation learning in both local and global contexts.

As shown in Figure 2, our framework introduces two complementary modules:

Domain-knowledge guided instance-level contrast. For each training instance, in addition to standard augmentations, we identify semantically similar samples using features extracted via domain-

specific signal processing. These nearest neighbours are treated as additional positives during contrastive training, helping to preserve clinically relevant relationships that conventional instance-based SSCL might overlook.

Domain-knowledge guided prototype-level contrast. To capture higher-order structure across the whole dataset, we apply unsupervised clustering to the domain-informed features of the training set, prior to training. Each cluster serves as a prototype representing a group of semantically related signals. During training, the network is encouraged to align each sample's representation with its corresponding prototype, thereby reinforcing consistency and robustness at a global level."

(2) enhancing the clarity of Figure 1.

To further illustrate the contribution, we have revised the original Figure 1 and split them to two figures. The updated Figure 1 illustrates the difference between traditional ("old-school") manual feature engineering and modern ("new-school") self-supervised contrastive learning (SSCL), and why the implicit domain expertise encoded in domain features is important, in terms of the shortcomings of "new-school" self-supervised contrastive learning. In particular, the figure emphasizes the issue that SSCL typically treats all non-identical samples as negatives, even when they may share important clinical similarities.

In addition, we revised the framework part (bottom panel) of the original Figure 1, which is now presented as Figure 2. The updated figure more clearly delineates the four key components of our proposed framework: (a) offline extraction and clustering of domain-informed features, (b) augmentation and shared encoding of input signals, (c) instance-level contrast guided by nearest neighbours in the domain feature space, and (d) prototype-level contrast based on domain-guided clustering. The would help to better understand how domain knowledge is integrated throughout the self-supervised learning process.

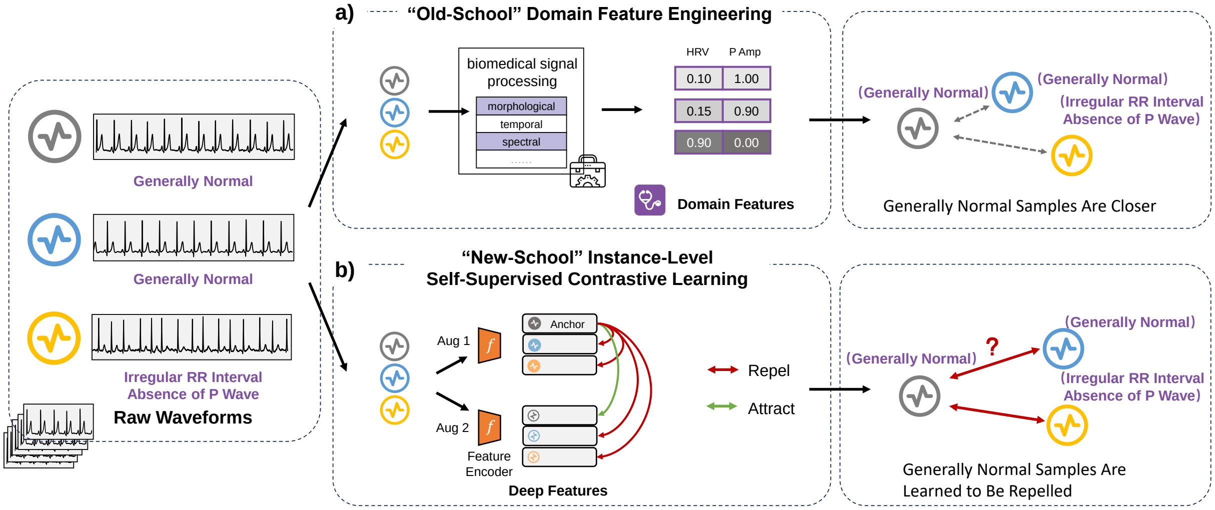

Figure 1: **Illustration of the pipelines of “old-school” domain feature engineering and “new-school” self-supervised contrastive learning (SSCL), for healthcare wearables.** Take electrocardiogram (ECG) as an example, a common wearable modality for detecting cardiovascular abnormalities. Notably, both pipelines function without necessarily requiring access to ground-truth labels. **a)** In the “old-school” pipeline, domain features such as heart rate variability (HRV), and the presence or amplitude of P-waves can be extracted using off-the-shelf biomedical signal processing toolboxes. These features are grounded in clinical knowledge and have long been used to support cardiovascular diagnosis. For instance, a normal reference ECG with stable RR intervals and clear P-waves would be grouped closer to another normal ECG with similar physiological characteristics, and distinctly separated from a abnormal ECG showing irregular rhythms or missing waveform components. **b)** In the “new-school” SSCL pipeline, representations are learned by contrasting augmented views of unlabeled data. Specifically, for each input signal, two augmented views (Aug 1 and Aug 2) are generated and passed through a shared encoder. The model is trained using a contrastive loss to pull the positive pair (the two augmentations) closer in representation space, while pushing them away from all other instances in the batch (treated as negative pairs), regardless of their physiological similarity. This could yield suboptimal representations. For example, a normal anchor ECG may be treated as equally dissimilar to both a generally normal sample and a clinically abnormal one, despite their differing clinical significance.

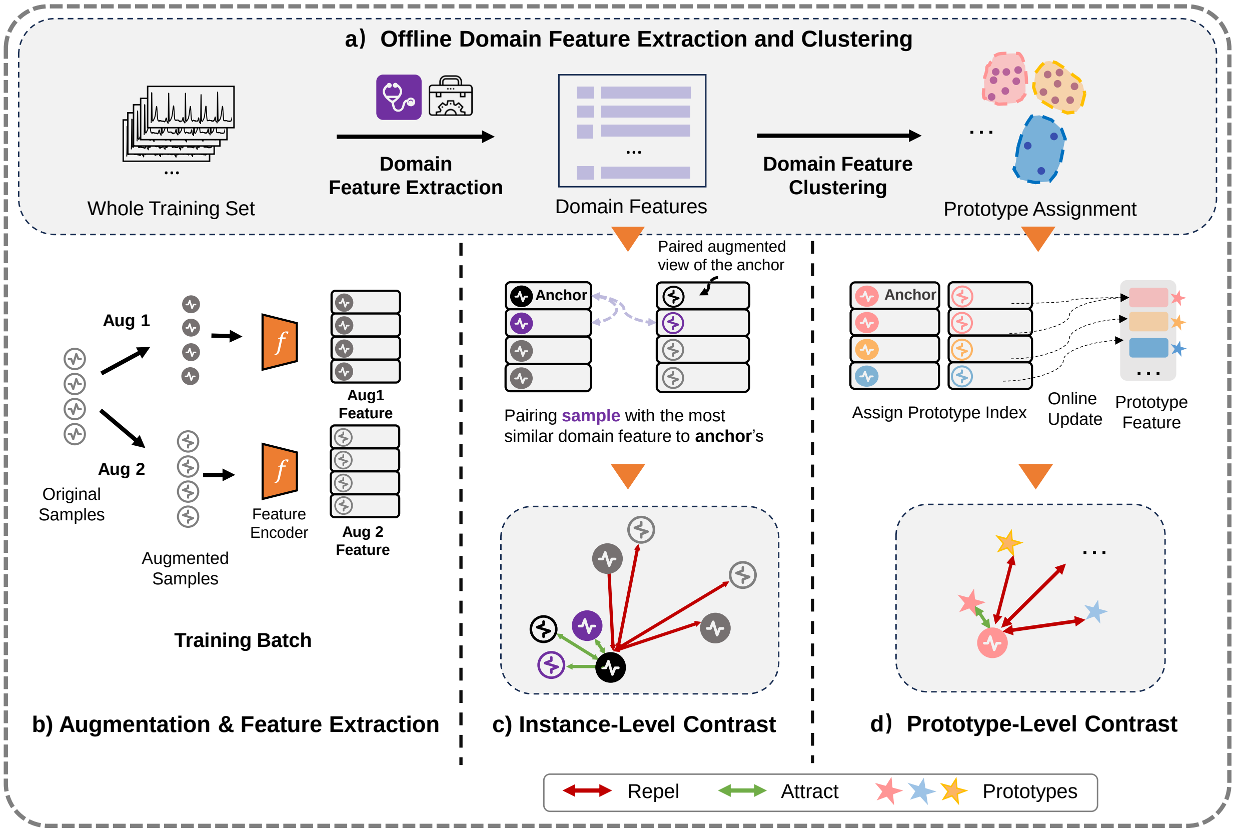

Figure 2: **Illustration of our domain-knowledge-guided self-supervised contrastive learning framework for decoding healthcare wearable time series data.** It incorporates medical domain expertise, which is implicitly "embedded" in "old-school" domain feature engineering, into a "new-school" self-supervised contrastive learning (SSCL) framework for decoding wearable waveforms. **a) Offline Domain Feature Extraction and Clustering.** Before training, we extract domain knowledge-informed bespoke features from all input training samples following domain-specific biomedical signal processing practices and toolboxes. These features are subsequently clustered via offline k-means to assign each sample to a domain-feature based prototype group. **b) Augmentation and Deep Feature Extraction.** During training, within each batch, each input signal is augmented twice and passed through a shared encoder to produce latent representations, following a standard instance-level self-supervised contrastive learning pipeline. **c) Instance-Level Contrast.** In addition to treating paired augmented views as a positive pair (marked in black), the nearest neighbour in the domain feature space is selected and its two augmented views (marked in purple) are included as additional positive. This enhances physiological consistency by preventing clinically similar samples from being treated as negatives. **d) Prototype-Level Contrast.** Subsequently, based on the preassigned prototype index from offline clustering, each prototype representation is updated online using an exponential moving average of the encoded features from its assigned samples. These prototypes capture and maintain the global structure of the training data. During training, each sample is encouraged to align with its corresponding domain-informed prototype, promoting consistency across the feature space.

C 1.2 — The method is divided into separate sections ("Overall Framework" and "Methods"), which creates confusion. These sections should be consolidated for coherence. The "Overall Framework" section, along with the figure, does not sufficiently explain the method. Key questions—such as how traditional ("old-school") features are combined with modern ("new-school") features, how domain-specific features are encoded, and how self-supervised learning (SSL) features are encoded—are only answered later in the "Methods" section. Including these details in the overview would improve clarity.

Reply: Thanks for pointing this out.

To address this, we have reorganized and consolidated the presentation of our method. Specially:
(1) Framework overview revision. We now provide a clearer and more intuitive overview of the entire framework in the revised Introduction and the newly structured "Overview of Proposed Method" (now positioned as the first subsection of the Results), emphasizing how "old-school" domain features are integrated into "new-school" self-supervised contrastive learning (SSCL).
(2) Adding key methodological components in Introduction. We explain how domain-specific features are extracted and utilized to guide both instance-level nearest neighbour selection and prototype-level contrastive learning. These points are now discussed directly in the Introduction with reference to Figure 2.
(3) Figure revision. The new Figure 2 complements this revision by visually delineating the four main components of our approach: (a) domain feature extraction and clustering, (b) signal augmentation and feature encoding, (c) instance-level contrast, and (d) prototype-level contrast.
(4) Reorganization of results. To maintain readability, we have intentionally left lower-level implementation details—such as loss formulations, normalization procedures, and training protocols—to the Methods section, with clear forward references provided for readers interested in the technical specifics. Additionally, ablation studies and hyperparameter experiments related to implementation (but not central to our key contributions) are now included in the Supplementary Materials to avoid overloading the main narrative and to ensure clarity.

Below are the revised Overview of Proposed Method:
Overall Framework. "We developed a novel domain-knowledge-guided SSL framework to bridge medical domain expertise (*"old-school"* domain feature engineering manner) with advanced deep learning techniques (*"new-school"* SSCL), for label-efficient wearable data interpretation. The overall framework consists of four stages: (a) offline domain feature extraction and clustering, (b) data augmentation and deep feature extraction, (c) domain-guided instance-level contrast, and (d) domain-guided prototype-level contrast, as illustrated in Figure 2.

(a) Offline Domain Feature Extraction and Clustering. Before training, we extract handcrafted features from all input training samples using domain-specific signal processing tools. These features are computed using open-source domain feature engineering pipelines and reflect established clinical heuristics. The extracted features are then clustered using offline k-means, assigning each sample to a prototype group based on domain-informed similarity. This clustering provides a coarse-grained organization of the training set, reflecting broader semantical groupings.

(b) Augmentation and Deep Feature Extraction. During training, each input time series undergoes two random augmentations (e.g., jittering, scaling, warping), producing two distinct but related views of the same signal. Both views are passed through a shared encoder network to extract latent representations. This follows the standard SSCL setup, where the model learns by contrasting pairs of representations across the batch.

(c) Domain-Guided Instance-Level Contrast. Conventional instance-level SSCL only considers

the augmentation from the same signal as positive pairs. In addition to this, we identify additional positive pairs using the similarity of domain features. Specifically, within each batch, based on similarity in the domain feature space, the nearest neighbour of the anchor signal is selected. The two augmented views of this neighbour sample are treated as additional positive, reinforcing the relationship between signals that are semantically similar but not augmented views of the same instance. This process mitigates the risk of treating semantically similar signals as negatives, which is a limitation of conventional instance-level SSCL.

(d) Domain-Guided Prototype-Level Contrast. To incorporate global structure, we use the offline cluster assignments from step (a) as a reference for prototype-level contrast. For each cluster, we maintain a prototype representation, which is updated during training using the exponential moving average of its members' latent features. During training, each sample is encouraged to align with its assigned prototype in the latent space. This ensures that the model preserves broader structure in the data distribution, helping to stabilize training and improve generalization across diverse physiological conditions.

For technical implementation details, including loss formulations, feature extraction procedures, and training protocols, please refer to the Methods Section."

C 1.3 — The accompanying figure is difficult to interpret:

- The color coding (e.g., many purple features) is ambiguous, as not all features mapped to the embedding space are clearly labeled.

- The varying sizes of the features and the lack of clear column correspondence to inputs and representations add to the confusion.

- Subfigure (b) lacks context—how is it used in the framework? Are there sorting constraints, and how are "hard negatives" addressed? The corresponding section should be explicitly referenced.

- Visual clarity is an issue (e.g., white text on light gray background is hard to read). A legend is missing. The figure should be revised for better clarity and effectiveness.

Reply: Thanks for the suggestion. We have updated the figure together in line with C 1.1, for better understanding. Please see as Figure 1 and Figure 2.

Specifically, original subfigure (b) was intended as a conceptual schematic; apologies for the confusion! We have now provided explicit context and references to its role within the framework. Especially in the Introduction,

Page 2 "As illustrated in the bottom panel of Figure 1, conventional SSCL typically treats two augmentations of the same instance as a positive pair, while all other samples in the batch are considered negatives—even if they may be physiologically similar."

Page 2 "conventional supervised learning methodologies for healthcare wearables have heavily relied on manually crafted features based on domain-specific knowledge, as shown in the top panel of Figure 1."

C 1.4 — The "Methods" section is placed at the end, which disrupts the logical flow. It would be more helpful to present the method before the results.

Reply: Thanks for pointing this out. Yes, we agree that placing the "Methods" section solely at the end does not facilitate reader understanding.

In response, and in line with C 1.2, we have merged the key methodological components from the original Methods section into a new Overview of Proposed Method subsection at the start of the Results

section. This revised overview outlines the overall framework and the critical steps associated with our main contributions, providing a more accessible and coherent explanation of the approach.

Likewise, as also noted in C 1.2, we have retained the detailed technical implementations—such as loss formulations, training protocols, and hyperparameter settings—in the dedicated Methods section at the end of the manuscript, in accordance with *Communications Engineering* formatting guidelines. Clear forward references are provided throughout the main text to help readers navigate between the conceptual overview and the corresponding technical specifics. Additionally, we have moved results that are not central to our contributions (e.g., ablation studies and hyperparameter sensitivity analyses) to the Supplementary Information to maintain clarity and focus in the main paper.

C 1.5 — How is it ensured that domain-specific features and SSL features are combined? The guided aspect of the approach needs clearer explanation as it the main concept of the submission and the details are presented only in the end.

Reply: Many thanks for this comment. We have substantially revised both the end of the Introduction and the beginning of the Results section (Overview of Proposed Method) to provide a clearer explanation of this guided mechanism, in line with C 1.1 and C 1.2.

To make it clear here, the domain features are offline extracted based on conventional domain feature engineering, which are subsequently used to guide the self-supervised contrastive procedure. In particular, they offer two types of guidance, building upon the conventional self-supervised contrastive learning, instance-level and prototype-level, as further illustrated in C 1.1.

C 1.6 — The authors claim that annotating wearable device data is costly but provide no concrete estimates or references to support this assertion. Including such data, along with examples of annotations in the supplementary material, would be beneficial.

Reply: Thanks a lot for the suggestion.

To clarify why annotating wearable device data is costly, we have expanded the introduction to explicitly describe the annotation process and challenges. Specifically, we highlight two common annotation approaches, which is added in the Supplementary Page 8 Section S5.1 :

(1) Direct Manual Annotation from Raw Data.

Expert clinicians manually inspect and annotate waveforms, which is labor-intensive, time-consuming, and requires specialized medical knowledge. For instance, annotating ECG or EEG recordings typically involves cardiologists or neurologists, respectively, carefully labeling abnormalities or distinct waveform patterns. Achieving consistent and accurate annotations generally necessitates multiple senior clinicians reviewing each recording, and to reach consensus between each other, further increasing resource demands [1].

(2) Annotation Using Additional "Gold-Standard" Devices.

Although raw waveforms from wearables are rich in information, obtaining explicit annotations often requires external reference equipment to establish reliable ground-truth labels. For instance, accurately annotating activity labels typically requires using external cameras as a reference, mostly coupled with crowdsourcing or expert labeling, to generate dependable ground-truth annotations. Notable examples include datasets like Capture-24 [2], where wearable signals are aligned and annotated using synchronized video recordings, to obtain reliable ground-truth data. Annotators typically require systematic training, and achieving consistent, reliable consensus among multiple annotators remains essential [2].

Apart from this, we also added some emphasize in the main paper as below,

Page 2 "However, in healthcare wearables, manually annotating large datasets, such as ECG or EEG signals, is both labor-intensive and costly [3], often requiring substantial medical domain expertise and, in some cases, additional expensive equipment serving as a "gold standard" (extended discussion are available in Supplementary Material Section S5.1). For example, sleep stage annotation on EEG [4] typically requires polysomnography, a comprehensive recording of physiological signals including brain activity (EEG), eye movement, and muscle activity. These recordings must be examined by trained technicians or sleep specialists, making the annotation process time-consuming and resource-intensive. This creates a significant bottleneck in scaling the use of deep learning for healthcare wearable applications, when translating the raw high-dimensional waveforms into actionable clinical insights."

C 1.7 — The authors state that SSL methods fail to align with semantic knowledge required for downstream applications but do not provide references or analysis to support this claim. Adding supporting evidence is necessary.

Reply: This is a very insightful point.

(1) Explanation of the original sentence.

The context of the original sentence was intended to highlight that many prior SSCL methods adopt sampling strategies that group positive pairs based on simplified assumptions—such as recordings from the same patient [5] or adjacent time frames [6]. However, these assumptions may not always hold in real-world healthcare settings. For instance, two ECG recordings from the same patient can differ substantially due to sudden cardiac events [7], while signals from different individuals may appear highly similar if both are healthy. Consequently, such pairing strategies may fail to capture the true clinical or physiological relationships required for downstream healthcare tasks.

We have revised the last two sentences, to "For instance, two ECG recordings from the same patient may vary significantly due to sudden cardiac events [7], while recordings from different patients might share similar characteristics if both are healthy. As a result, following such explicit positive pair grouping policy sometimes may fail to align with the semantic knowledge needed for actual downstream healthcare applications. "

(2) Positive rate of similar domain features based on downstream annotations.

On the other hand, we measured, with the availability of downstream annotations, whether the instances with similar domain features would belong to the same category or not. We compared the domain-knowledge-based features (extracted from domain feature engineering) and the feature learned from SimCLR. Specifically, we randomly sampled 100 mini-batches (batch size = 1,024) from the CPSC dataset and computed the positive rate—the probability that the nearest neighbors in the feature space share the same class label. Lower similarity orders correspond to closer neighbors. As shown in Figure 3, domain-knowledge-based features consistently yielded higher positive rates among top-ranked neighbors, indicating better alignment with class-relevant semantics. In contrast, features learned via SimCLR tended to separate semantically similar samples, highlighting the limitations of conventional SSCL in preserving meaningful clinical relationships.

Further to the above observation, one key reason why conventional self-supervised contrastive learning (SSCL) may fail to align with semantic knowledge is that it typically relies on instance discrimination, where all non-identical samples are treated as negatives—regardless of their clinical or physiological similarity. This issue is also conceptually illustrated in Figure 1.

In practice, this often results in semantically similar samples—such as those from the same downstream class—being pushed apart during training. These unintended negative pairings are commonly

referred to as false negatives [8], and they undermine the ability of SSCL methods to preserve class-relevant structure in the learned representation space. In line with our observation in Figure 3, domain features guided pairs can help resolve the false negative issue to some extend.

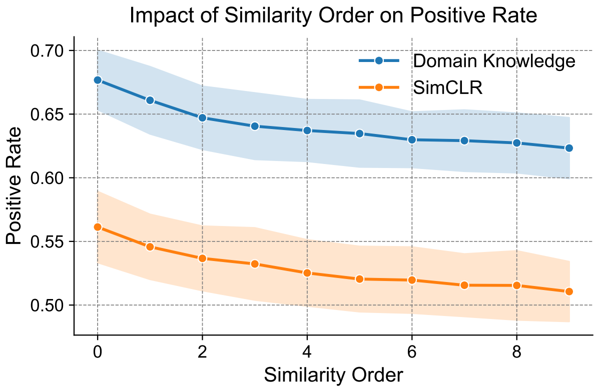

Figure 3: Positive rate across similarity orders of features, comparing domain feature engineering based features and SimCLR embeddings. We randomly sample 100 times with a batchsize as 1024 from the training samples of CPSC, and calculate. Each point represents the mean positive rate across trials, with shaded regions denoting the standard deviation. Lower similarity orders correspond to feature-space neighbors closest to a reference instance. Domain knowledge–based features consistently yield higher positive rates at lower similarity orders, suggesting improved semantic alignment and class structure preservation compared to SimCLR representations.

C 1.8 — While the authors reference SSL methods and healthcare applications, the cited works (from 2021 and 2022) may not fully reflect the state-of-the-art. Are there more recent studies?

Reply: This is a very good point. We try to address you concerns in the following two aspects.
(1) Extended Discussion. We have included more recent self-supervised learning methods, by incorporating or changing to more recent works.

Page 2 "Various strategies have been proposed to find/create appropriate pairs in SSCL, including effective data augmentation techniques, such as adding noise or temporal flipping, that create different augmented views of the same instance [9]. Regarding the former, there are several works that aim to generate domain-inspired augmentation strategies to enhance representation learning [10, 11]. However, these methods are often sophisticatedly tailored to specific wearable modalities and lack generalizability across different signal types, due to inherent heterogeneity among modalities. The other line of research resorts to sampling strategies that group positive pairs based on shared medical concepts [12, 6]. Typical examples of the latter include treating records from the same patient [5] or from nearby time frames as positive pairs [6]. However, these approaches often introduce inductive biases that may conflict with the underlying medical semantics of the data. For instance, two ECG recordings from the same patient may vary significantly due to sudden cardiac events [7], while recordings from different patients might share similar characteristics if both are healthy. As a result, following such explicit positive pair grouping policy sometimes may fail to align with the semantic knowledge needed for actual downstream healthcare applications."

Additionally, in line with C 1.22, we have revised the citations of previously referenced works, by replacing outdated arXiv references and correcting the publishing years where applicable.
(2) Extended Experiments. In terms of experiments, we added the comparison from one strong self-supervised contrastive baseline SwAV[13], more recent self-supervised contrastive work AMCL[14] and SoftIns [15], as well as recent general-purpose time series foundation model (with large scale self-supervised pretraining on general time series) MOMENT[16] and Chronos[17]. These provide a more up-to-date and comprehensive benchmark against our proposed approach. This ensures that our findings are well-situated within the current landscape of SSL research.

Page 6 "To highlight the superiority of our proposed framework, we compared it against a comprehensive suite of existing self-supervised learning methods. These include general-purpose contrastive learning frameworks such as SimCLR [18], BYOL [19], MoCo [20], NNCLR [21], TS [22], SwAV [13], and AMCL [14], all adapted for time series data. We also evaluated domain-specific baselines, including CLOCS [6], originally developed for ECG-based self-supervised learning, and TFC [12] and SoftIns [15], both designed for general time series modalities. Additionally, RNC [12] was employed to rank time series representations based on domain features, providing a comparative approach for evaluating how domain knowledge can be incorporated into self-supervised learning. For fair comparison, without explicitly mention, all methods were implemented using a ResNet18 [23](1d version) backbone to derive deep features."

Page 6 "Furthermore, we incorporated an additional group of comparisons within the broader **self-supervised learning** landscape. Recent advances in general-purpose time series foundation models [24] have demonstrated the potential to handle diverse temporal signals through large-scale pretraining on heterogeneous datasets [17, 16]. To benchmark our framework against this paradigm, we evaluated the representation quality extracted from two representative encoder-decoder-based foundation models: MOMENT[16] and Chronos[17]. These models provide pretrained encoders capable of generating embeddings across a wide range of time series modalities. "

Table 1: **Comparison results based on self-supervised learning.** To test the efficacy of the downstream knowledge learned during self-supervised learning, we froze the backbone as a feature extractor, and applied KNN and a linear classifier for supervised training. We compared the class-average F1 on the test subset based on the model with the best F1 value on validation subset. Each method was run with five different random seeds to assess the performance. We reported the mean ± standard deviation of the five results. **Bold** indicates the best result, and Underline indicates the second best result.

Methods	KNN				Linear Probing			
	ECG	EEG	IMU	PPG	ECG	EEG	IMU	PPG
Fully Sup.	-	-	-	-	0.641±0.02	0.658±0.01	0.484±0.05	0.520±0.04
Domain Feat.	0.489±0.05	0.392±0.02	0.470±0.05	0.373±0.04	0.509±0.04	0.548±0.04	0.422±0.06	0.377±0.05
SimCLR [18]	0.427±0.03	0.574±0.02	0.393±0.04	0.395±0.10	0.532±0.07	0.617±0.03	0.433±0.03	0.414±0.05
BYOL [19]	0.380±0.05	0.445±0.09	0.418±0.10	0.243±0.12	0.428±0.04	0.431±0.10	0.449±0.09	0.326±0.08
MoCo [20]	0.450±0.02	0.568±0.04	0.408±0.04	0.368±0.05	0.524±0.05	0.594±0.08	0.425±0.01	0.365±0.04
NNCLR [21]	0.439±0.04	0.511±0.04	0.383±0.06	0.394±0.07	0.517±0.05	0.555±0.03	0.387±0.04	0.355±0.06
TS [22]	0.474±0.05	0.580±0.10	0.449±0.03	0.395±0.04	0.549±0.10	0.627±0.03	0.501±0.06	0.452±0.03
SwAV [13]	0.452±0.10	0.561±0.05	0.438±0.04	0.343±0.02	0.510±0.08	0.638±0.10	0.463±0.07	0.406±0.03
AMCL [14]	0.402±0.06	0.570±0.07	0.413±0.02	0.301±0.05	0.503±0.04	0.626±0.05	0.447±0.05	0.419±0.07
CLOCS [6]	0.488±0.01	0.581±0.06	0.451±0.05	0.376±0.05	0.537±0.03	0.642±0.05	0.483±0.05	0.451±0.06
TFC [12]	0.405±0.03	0.543±0.03	0.267±0.04	0.383±0.04	0.411±0.02	0.571±0.07	0.307±0.01	0.401±0.04
SoftIns [15]	0.481±0.04	0.539±0.03	0.420±0.05	0.337±0.05	0.557±0.10	0.602±0.03	0.498±0.08	0.382±0.06
RNC [25]	0.478±0.04	0.504±0.08	0.465±0.01	0.342±0.02	0.550±0.03	0.560±0.02	0.517±0.05	0.424±0.05
MOMENT [16]	0.443±0.03	0.464±0.02	**0.517**±0.02	0.366±0.01	0.514±0.03	0.456±0.01	0.522±0.02	0.411±0.03
Chronos [17]	0.325±0.03	0.452±0.04	0.445±0.04	0.387±0.05	0.301±0.08	0.406±0.06	0.481±0.06	0.410±0.10
Ours	**0.509**±0.04	**0.591**±0.07	0.465±0.02	**0.420**±0.04	**0.574**±0.03	**0.643**±0.03	**0.526**±0.04	**0.499**±0.02

C 1.9 — Furthermore, the methods compared in Tables 2, 3, 5, and 6 are general SSL frameworks rather than domain-specific approaches for healthcare or wearable devices. Including comparisons to more relevant methods would strengthen the submission.

Reply:

Many suggestions for the suggestions. We have expanded the original manuscript by the following three aspects:

(1) Inclusion of more time-series specific methods: Original implementations include CLOCS[6], originally developed by our group, specifically designed for self-supervised ECG classification, and TFC[12] targets alignment by aligning temporal and frequency level representations for time-series inputs. In addition, we implemented SoftIns from Lee *et al.* [15], which assigns continuous similarity weights to positive and negative pairs based on their original signal-space distance, characterized using Dynamic Time Warping.

(2) Comparison with general-purpose time series foundation model in the main paper:

We have incorporated evaluations of two recent state-of-the-art general-purpose time series foundation models—MOMENT[16] and Chronos[17]—within the main paper. These models are pretrained on large-scale, heterogeneous time series datasets and represent the latest advances in generalizable time series representation learning.

Results regarding **(1)** and **(2)** are presented in Table 1 (Table 2 in the main paper).

(3) Comparison with wearable-specific foundation model in the Supplementary: We additionally include results in the Supplementary comparing our method against modality-specific pretrained models designed for waveforms [26, 27]. While such models are often tuned for particular sensor types or clinical

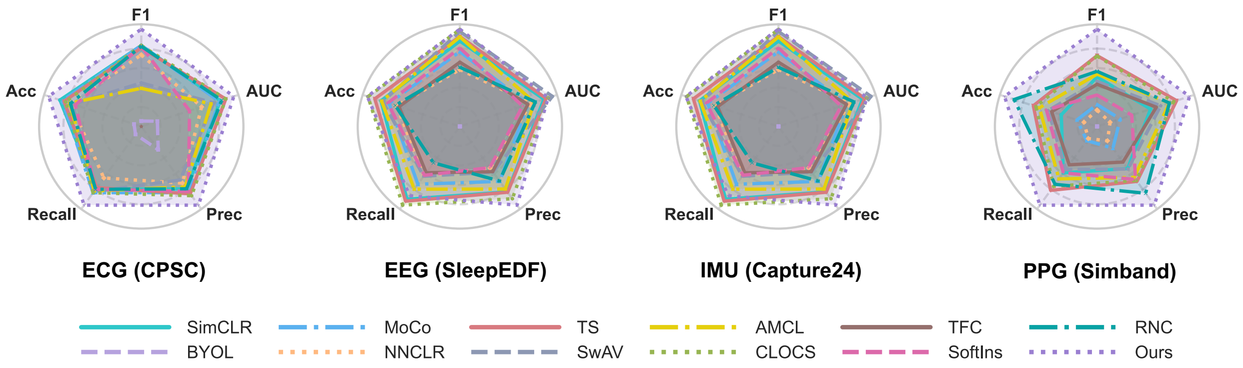

Figure 4: **Performance comparison in terms of class-average F1, Accuracy (Acc), AUC, Recall, and Precision (Prec).** We compared the performance on the test subset, based on the model with the best F1 score on the validation subset. The values are normalized by the deviation from the best performance for each respective metric.

tasks and thus may not generalize across modalities, we still evaluated them to provide additional insight.

In detail, we compared with two wearable-modality-specific foundation models (ECGFM [26] for 12-Lead Diagnostic ECG and PaPaGei [27] for PPG). The results, measured in terms of class-average F1 score, are summarized below, with our model pretrained using self-supervised learning.

Table 2: **Comparison with wearable-modality-specific foundation models. We evaluate our self-supervised pretrained model against ECGFM [26] and PaPaGei [27], two foundation models tailored for 12-lead ECG and PPG signals, respectively.** Performance is reported as class-average F1 score using both KNN and linear probing.

Method	KNN			Linear Probing		
	ECG (CPSC)	ECG (CinC17)	PPG (SimBand)	ECG (CPSC)	ECG (CinC17)	PPG (SimBand)
PaPaGei[27]	-	-	0.335	-	-	0.417
ECGFM[26]	0.511	0.565	-	0.557	0.582	-
Ours	0.509	0.571	0.420	0.574	0.540	0.499

For ECGFM, we used the publicly available pretrained model[1] and adapted our data by duplicating the single-channel ECG signals to 12 channels to match the model's input requirements. For PaPaGei, we followed the official implementation pipeline[2] for PPG representation extraction and evaluation.

As shown in Table 2, ECGFM performs competitively on ECG datasets and occasionally surpasses our method, particularly on CinC17. On the other hand, PaPaGei also shows reasonable performance on PPG signals.

Please noted that ECGFM was pretrained on the CPSC dataset in a self-supervised manner, and since the exact pretraining setup is not fully disclosed, it is possible that test data may have been included during pretraining.

[1]https://github.com/bowang-lab/ECG-FM

[2]https://github.com/Nokia-Bell-Labs/papagei-foundation-model

It is also important to note that direct comparisons with these large foundation models are not entirely equitable, due to differences in model size, training scale, and modality-specific design. Our method, by contrast, is lightweight and generalizable across multiple wearable modalities. Incorporating our domain-guided strategies into future foundation model development may enhance both their interpretability and clinical applicability.

C 1.10 — A direct connection between the proposed approach and applications like "physical activity monitoring," "wellness assessments," and "fitness tracking" is lacking. How do experiments with ECG, EEG, and IMU translate into these applications? Although Table 1 lists tasks, it is neither referenced nor discussed. Are all tasks classification tasks? Clarification and integration into the narrative would improve the manuscript.

Reply: Many thanks for the comments. We have revised to clarify the connection between each dataset, its corresponding modality, and the real-world application it represents.

The updated text and Table are as below, "To assess the feasibility and generalizability of our framework, we conducted experiments across four types of wearable sensing modalities: electrocardiogram (ECG), electroencephalogram (EEG), inertial measurement unit (IMU, focusing on 3-Axis Accelerometer), and photoplethysmography (PPG). All downstream tasks are framed as classification problems, aligning with common healthcare applications associated with each modality, such as physical activity monitoring, cardiac event detection, and sleep stage assessment."

Table 3: **Summary of datasets, modalities, and corresponding real-world applications.** This table outlines the datasets used in our experiments, the type of wearable modality they represent, and the associated downstream classification tasks. These tasks, based on the expert annotations provided with each dataset, span clinically and behaviorally relevant applications, including cardiac rhythm classification (ECG, PPG), sleep stage detection (EEG), and physical activity recognition (IMU). All tasks are formulated as multi-class classification problems.

Dataset	Modality	Downstream Task	# Class	# Sample	# Channel	Bandpass	Win (s)	Freq (Hz)
CinC17[28]	ECG	Cardiovascular Disease Classification	4	8528	1	0.5-40	10	125
CPSC[29]	ECG	Rhythm/Morphology Abnormality Identification	9	6877	1	0.5-40	10	125
MIMIC-III-WDB[30]	ECG	-	-	111619	1	0.5-40	10	125
SleepEDF[4]	EEG	Sleep Staging	5	41509	2	0.4-30	30	100
Capture24[2]	IMU	Activity Recognition	6	45553	3	-	10	100
Simband[31, 32]	PPG	Cardiovascular Disease Classification	4	7590	1	0.5-20	10	50

C 1.11 — The authors claim their approach significantly improves generalization for rare but important health signals but do not provide corresponding analysis or results. This claim needs to be supported with evidence or clearly pointed out in the results.

Reply: This is a very constructive point. We have added the following additional analysis in the Supplementary.

As shown in the new Figure 5, we plot the per-class accuracy improvement of our method compared to SimCLR, against class size (sorted from most to least frequent class). The black line indicates the number of training samples per class, while the green bars represent the change in classification accuracy achieved by our approach. This analysis reveals that our method offers more stable and often larger

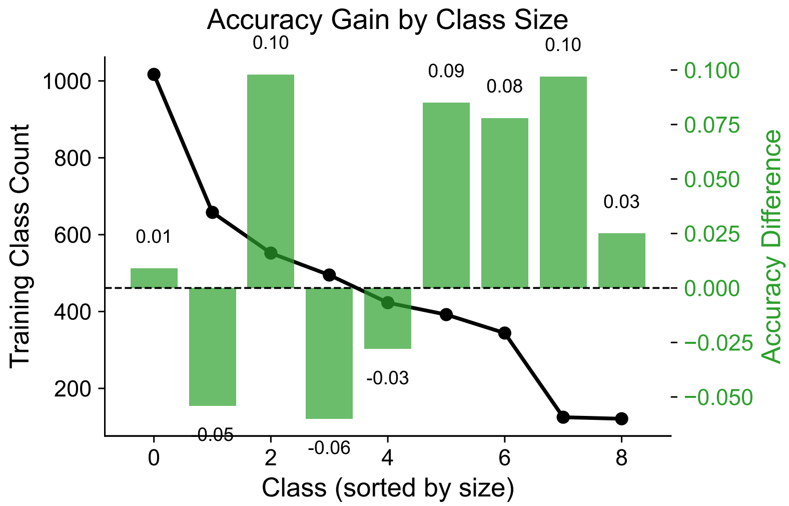

Figure 5: **Per-class accuracy improvement compared to SimCLR, plotted against class size (sorted from largest to smallest)**. The line represents the number of training samples per class, while bars indicate the change in accuracy achieved by our method over SimCLR. Classes with smaller sample sizes generally exhibit more stable improvements, while gains in larger classes are more variable.

improvements for classes with fewer training samples, whereas performance gains for more common classes are more variable. This result confirms that domain knowledge guided contrast can be especially beneficial for underrepresented classes, often the ones associated with rare but critical clinical conditions.

C 1.12 — - It is unclear how waveforms are encoded. This information should be added.

- The effect of the number of neighbors chosen on performance is not addressed.

- The application of semi-supervised methods is ambiguous. Do the authors create pseudo-labels or merely add a supervised loss on top? This should be clarified in both the method and experimental sections.

Reply: Thanks for raising these thoughtful points! Regarding your concerns:

(1) Encoding of waveforms

We now explicitly clarify in the revised Introduction and Methods sections that all input waveform segments are first augmented and then passed through a feature encoder to extract latent representations. "During training, each input time series undergoes two random augmentations (e.g., jittering,

scaling, warping), producing two distinct but related views of the same signal. Both views are passed through a shared encoder network to extract latent representations. "

These feature encoder was explicitly mentioned as ResNet1d18 later in Experimental Setup. "For fair comparison, without explicitly mentioning, all methods were implemented using a ResNet1d18 backbone to derive deep features."

(2) Effect of the number of neighbors in domain-guided instance-level contrast

This is an excellent point. The number of neighbours is a good point to investigate and explain. As suggested in Figure 7, we plotted the positive rate across similarity orders based on domain features. The results indicate that as the similarity order increases (i.e., neighbors become less similar in the feature space), the likelihood of them belonging to the same class decreases. This suggests that incorporating more neighbors may introduce semantically inconsistent or even contradictory supervision signals.

In our ablation experiments, we evaluated the impact of varying the number of neighbors ($k = 1, 3, 5$). We found that performance generally plateaued or slightly declined beyond $k = 1$. For example, in ECG linear probing, the F1 scores were 0.574, 0.575, and 0.559, respectively; while for EEG, the scores were 0.642, 0.634, and 0.625. These findings indicate that adding more neighbors does not consistently improve performance and may instead introduce noise.

Based on this, we adopted the top-1 strategy as our default setting, given its simplicity, semantic consistency, and empirical robustness across modalities.

(3) Semi-supervised learning protocol Pure supervised loss on the labeled subset. We have revised the original text to "In the **semi-supervised** setup, we randomly sampled a proportion (5%, 10%, and 20%) of the full training datasets (CinC17 and CPSC) and fine-tuned the entire model using a supervised loss applied only to the available labeled data."

C 1.13 — The discussion of related work is missing. Recent advances in general SSL and representation learning for wearable devices, as well as semi-supervised frameworks (e.g., FixMatch), should be included as a separate section.

Reply: Thanks for the suggestion. We have added this discussion in the Section S5.2 Extended Related Work of Supplementary.

"Faced with the challenges of annotating the waveforms streamed from wearables, emerging direction in healthcare wearables is to perform label-efficient decoding of healthcare wearables, minimizing the need for labels whilst learning good representations from these high-dimensional waveforms [3].

Self-Supervised Learning. Self-supervised learning (SSL) has become a dominant strategy for label-efficient learning in healthcare wearables. Among SSL methods, contrastive learning has been particularly popular [18]. It trains models to pull together representations of positive pairs (typically different augmented views of the same instance) while pushing apart negative pairs (different instances). This discriminative pretraining objective has been shown to encourage the model to learn meaningful and generalizable feature representations without manual supervision. However, as discussed in the main paper, these conventional instance-level contrast may not learn semantically meaningful representations.

In healthcare wearables, (or more generally, the time series domain), several advances have been made in self-supervised contrastive learning, identified across segment-, temporal-, individual-, and medical concept-level strategies. At the segment level, much of the focus has been on curating domain-specific/generic augmentations for waveform signals. Representative examples include optimizing the temporal-frequency representation consistency in [12], and some domain-specific augmentations [10]. Given the inherently sequential nature of biosignals, several methods further exploit temporal relationships [7, 15] to define contrastive views. For instance, Lan *et al.*[7] introduced a stationarity-aware

contrastive learning strategy that detects and preserves abrupt changes across neighboring frames. However, these approaches typically require access to long continuous recordings, which are not always available in wearable datasets collected under specific paradigms. Moreover, within-subject sequences can span varying physiological or pathological states, complicating identity-based consistency.

Beyond segments and timestamps, individual-level contrastive learning aims to preserve subject-specific characteristics across recordings. Sangha *et al.* [33] and Abbaspourazad *et al.* [5] showed that identity-aware contrastive learning improves representation quality and outperforms naive segment-level strategies. Still, this line of work assumes that multiple recordings are available per subject, which is often not the case in wearables. Lastly, some recent efforts have incorporated external modalities, such as paired clinical notes, to guide contrastive representation learning. Representative examples include recent ECG-Text contrastive learning framework proposed by Yu *et al.* [34]. Such approaches, while promising, rely on the availability of well-aligned auxiliary data, which many wearable modalities may lack, limiting their general applicability.

Time Series Foundation Models. Recently, self-supervised learning strategies have been extended to the training of large-scale models on diverse and heterogeneous time series datasets, giving rise to time series foundation models [24]. These models aim to learn general-purpose representations that can be transferred across a wide range of downstream tasks and domains. In the broader time series domain, several foundation models have been proposed [35, 16, 17], trained on large-scale corpora spanning domains such as finance, weather, traffic, etc. However, most of these pretraining datasets cover little to no medical (or even healthcare wearable) data. Given the fundamental differences in dynamics, noise characteristics, and clinical semantics between healthcare waveforms and non-medical time series, the applicability and effectiveness of these general-purpose foundation models in healthcare wearables remains largely unexplored and unvalidated.

On the other hand, several wearable-modality-specific foundation models have recently been proposed for wearable signals, such as PaPaGei [27] and Accelerometer Foundation Model [36]. These models have demonstrated strong representation learning capabilities on unseen data within the same modality. However, their pretraining paradigms often rely on modality-specific designs[36, 27] as the key contributions, limiting their applicability across diverse wearable sensing modalities.

That said, the development of full-scale foundation models is beyond the scope of this work. Instead, our focus is on advancing self-supervised contrastive learning strategies that are inherently generalizable across different wearable modalities. Importantly, we aim to provide practical guidance on how "readily available" medical knowledge can be incorporated into the self-supervised pretraining process, for training wearable foundation models, which is one of our future directions.

Semi-Supervised Learning. Compared to self-supervised learning, semi-supervised learning offers another promising strategy for label-efficient decoding of healthcare wearables by leveraging a small amount of labeled data alongside a large pool of unlabeled signals. A straightforward and simplistic approach is to integrate supervised training on the labelled subset, coupled with self-supervised learning over the whole set. Compared to self-supervised learning, more advanced strategies incorporate some surrogate objectives, such as pseudo-labeling or consistency regularization, to allow the model to benefit from both labeled guidance and unlabelled training signals.

These have been applied in wearable sensing modalities. Pseudo-labeling is a technique in which model predictions on unlabeled data are treated as temporary ground-truth labels for training. The key idea has been applied to semi-supervised learning of ECG [37] and EEG [38] signals. Another popular direction is consistency regularization, which assumes that model predictions should remain stable under input perturbations or augmentations. Among these, FixMatch [39], a widely used method, applies weak

and strong augmentations to the same input and trains the model to produce consistent predictions. This has been applied and extended in wearable contexts like accelerometer based activity recognition [40]. "

C 1.14 — Additional ablation studies are needed:
- The influence of cluster-specific temperature parameters during training.
- The impact of the progressive application of prototypical contrastive learning (e.g., what happens if it is applied with a fixed weight of 1?).

Reply: Thanks, we have added additional experiments and below are the results. As these details are not central for key contributions (only introduced in the end Methods section), and for better flow, we placed this Table in Supplementary.

As shown in Table 4, the experiments regarding the influence of temperature-specific parameters are illustrated based on the comparison between **a** and **e**. On the other hand, the impact of the progressive prototypical contrastive learning are shown based on the comparison between **b** and **e**.

In detail, (1) comparing **a** with **e**: assigning a cluster-specific temperature to each prototype was inspired by previous work [41] As shown in Table 4, using a cluster-specific temperature yields better performance, likely due to the improved balanced concentration across clusters.

(2) comparing **b** with **e**: Inspired by prior work [41], which applies warm-up training before introducing prototype-level contrastive learning, we also adopt a progressive scheduling strategy for the prototype contrastive loss. As shown in Table 4, applying both instance-level and prototype-level contrastive objectives from the very beginning (comparing **b** with **e**) leads to suboptimal performance. This might be due to reason that the prototype representations at the very initial point may not be good enough (only updated with a small number of samples). Forcing alignment of each instance to their positive pair and its corresponding prototype too early could destabilize training and hinder convergence.

Table 4: **Additional experiments on CPSC (ECG) dataset.** This includes ablation studies of different framework components and sensitivity studies of the clustering number.

	Version	F1
a	w/o temperature-wise schedule	0.563
b	w/o progressive	0.522
d	# Proto k=32	0.569
e	# Proto k=128 (Ours)	0.574
f	# Proto k=256	0.572

C 1.15 — BSCE Usage: The authors mention using BSCE but do not explain its importance. Is it only used for downstream classification tasks? This needs clarification.

Reply: Thanks for mentioning this. We applied BSCE [42] (Balanced Softmax Cross Entropy) only for downstream evaluation. This is a simple yet powerful loss function to tackle class imbalance issue,

We have added additional contents regarding this.

Page 15 "Given the imbalanced nature of most datasets, we employed Balanced Softmax Cross Entropy (BSCE)[42] to handle class imbalance. Without loss of generality, this was applied for the training of all downstream experiments. "

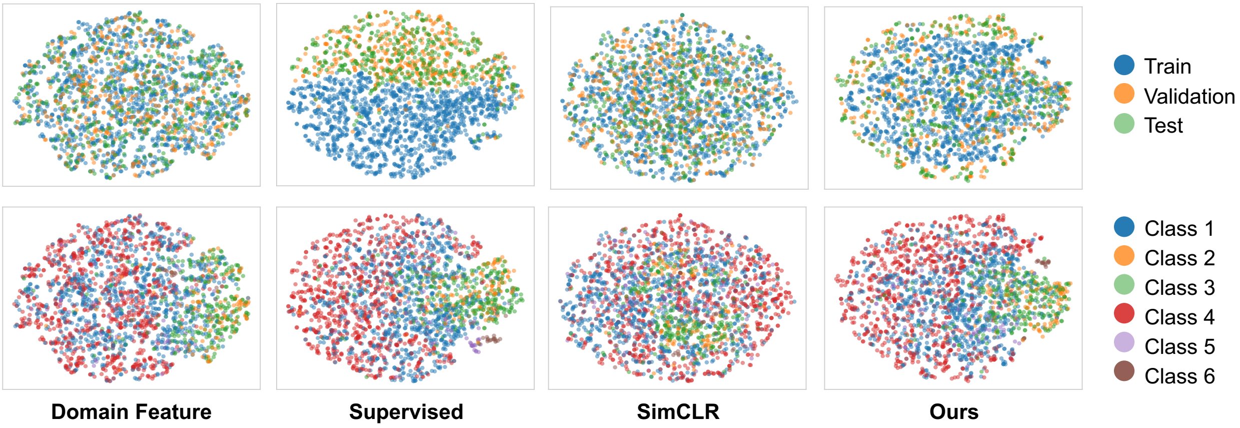

Figure 6: **t-SNE visualizations of domain features, features extracted from end-to-end supervised learning, from SimCLR and from our method, respectively.** Each point corresponds to a sample from the Capture24 dataset. Each column represents the same plots, with points colorized by different manners. In the top panel, each sample points were colored by data split (train/validation/test), whilst in the bottom, shaped by class label.

C 1.16 — Unexpected Results in Table 2: Linear probing with full supervision for IMU is significantly lower than for self-supervised training. Why does the "domain-guided" approach perform better here? What biases does it mitigate?

Reply: Many thanks! This is a very good point which also raised our attention during the preparation of this work.

We suggested that, Page 6 "Our method demonstrates superior performance compared to other SSL methods and even outperforms fully supervised models in certain cases, particularly with IMU data, where it achieves a class-average F1 score of 0.526 compared to 0.484. This improvement is likely attributable to our domain knowledge-guided approach, which helps mitigate the effects of inter-subject variability, especially in physical activity data, where individual movement patterns can differ significantly [2]."

We have shown additional qualitative analysis based on this,

Page 8 "we included a t-SNE visualization (Figure 6) comparing domain features, features from end-to-end supervised learning, those learned by SimCLR, and those learned by our method, from the whole dataset of Capture24. It can be observed from the first column, that the domain knowledge based features are evenly distributed between training/validation/testing subjects. In contrast, features learned via end-to-end supervised training (second column) exhibit clear clustering based on training and validation/test splits. This suggests that the model may have overfitted to subject-specific characteristics, such as individual wearing patterns or sensor noise, which are not directly related to activity related semantic information.

As shown in the third and fourth column, SimCLR and Ours, both approaches produce more evenly distributed feature representations across training, validation, and test sets. SimCLR performs instance-level contrast, which implicitly push away the representations of the instances from the same subject. This helps to avoid learning those subject-specific characteristics as biases. However, the learned representation are not semantically discriminative enough. As observed from the plot of second row, third column, points from different classes get more mixed compared to other plots of the same row. Our

method goes a step further, by using domain features to guide positive pair selection. This helps the model focus on learning semantically meaningful patterns rather than unrelated differences between individuals."

We have clarified this point in the revised manuscript.

C 1.17 — The plots in Figure 4 appear redundant, as they add no new information to the manuscript. Consider removing them to streamline the presentation.

Reply: Apologies for the confusion, The original intent of the original Figure 4 (now Figure 5) was to illustrate that our model learns discriminative features while maintaining strong generalization to unseen subjects—specifically by avoiding overfitting to subject-specific characteristics.

And thanks for the constructive point raised in C 1.16, in line with this, we have replaced the t-SNE plot as in Figure 6 to provide a clearer visualization using updated t-SNE plots. These now directly compare feature distributions from domain-feature-engineering based, supervised, SimCLR, and our methods. Hope it can facilitate understanding.

C 1.18 — Repetitive Sentence in "Overall Framework" Section: - The sentence *"…improve downstream validation performance, significantly improving downstream validation performance"* is redundant and needs to be revised for clarity and conciseness.

Reply: Thank you. This has been corrected.

C 1.19 — Word Choice in "Overall Framework" Section: - Replace *"leveraged"* with *"leverage"* for grammatical consistency.

Reply: Thank you. This has been rectified.

C 1.20 — Augmentations: - The authors follow prior works (e.g., 6, 11) on time series data, but the listed augmentations appear generic. Are there domain-specific augmentations tailored for this type of data that could be considered?

Reply: Thanks for pointing this out. - Domain specific augmentations would be a very interesting topic to explore.

Indeed, several studies emphasize the importance of carefully selecting and combining augmentations to improve contrastive learning performance. For example, SimCLR (the pioneering work of self-supervised contrastive learning [18]) demonstrates that the composition of data augmentations can significantly impact the learned representations. This finding has been extended to wearable sensing modalities such as ECG [6] and EEG [43]. These works collectively show that the optimal set of augmentations often depends on the specific dataset and application.

In contrast, whereas domain-specific features have traditionally been central to conventional biomedical signal-processing pipelines, domain-oriented data augmentation strategies remain less unified. Still, various research efforts have explored applying domain knowledge to mitigate data scarcity [3]. For example, Gopal *et al.* [10] mapped multi-lead ECGs to Vectorcardiogram (VCG) space, then applied spatial augmentations (e.g., rotation and scaling) in three-dimensional space before back-projecting the transformed signals to the ECG space.

In our study, we acknowledge the potential advantages of domain-specific augmentations; however, implementing them effectively across diverse sensing modalities is non-trivial and requires careful, modality-specific design, compared to leveraging domain-specific features that can be easily extracted by off-the-shelf toolboxes. Therefore, out of simplicity and consistency, we employ generic and consistent time-series augmentations across different sensing modalities, and to highlight our primary contribution—combining "old-school" domain-specific features for pair selection with a "new-school" self-supervised contrastive learning framework.

We have added a brief discussion in the main text to emphasize this rationale.

Introduction "Various strategies have been proposed to find/create appropriate pairs in SSCL, including effective data augmentation techniques, such as adding noise or temporal flipping, that create different augmented views of the same instance [9]. Regarding the former, there are several works that aim to generate domain-inspired augmentation strategies to enhance representation learning [10, 11]. However, these methods are often sophisticatedly tailored to specific wearable modalities and lack generalizability across different signal types, due to inherent heterogeneity among modalities."

Experimental Settings "Although some recent work has explored domain-specific augmentations for certain modalities (e.g., spatial transformations of ECG using vectorcardiography [10]), designing such strategies for diverse wearable signals is challenging and often requires modality-specific tailoring. In our study, we adopted a consistent, modality-agnostic augmentation pipeline to maintain simplicity and better highlight the effect of our domain-knowledge-guided contrastive learning framework."

C 1.21 — Section Naming: - The title of the "Methods" section suggests a combination of existing approaches rather than presenting the proposed method. Consider renaming it to better reflect its content, such as "Proposed Methodology" or "Methodological Framework."

Reply: Thanks a lot! To address this point, we have named the subsection within the Results part as Overview of Proposed Method to provide a high-level summary early in the paper. Meanwhile, we have retained the original Methods section for presenting technical details, in line with the editorial guidelines of *Communications Engineering*.

C 1.22 — Citations: Verify citation details for conferences such as ICLR and NeurIPS, as Google Scholar often lists incorrect publication years. For example, TS is published at ICLR 2023, not ICLR 2022.

Reply: Thank you! - we have updated the references, especially correcting those used to be Arxiv, and those with wrong year.

C 1.23 — Duplicate Tables: Tables 5 and 6 are identical and should be revised or removed to avoid redundancy.

Reply: Apologies and thanks, this has been corrected.

Reviewer 2

C 2.1 — Healthcare Wearables: authors claim a Unified framework for varied types of healthcare wearables. They should down-scale their claims since they work on four datasets (EEG, ECG and Activity Recognition) which are not necessarily representative of healthcare (sub) domains and applications within.

Reply: Thanks for the comment.

We agree that "unified" could imply broader generalizability than is currently demonstrated. Our intention is to emphasize that the proposed framework is applicable across a diverse set of healthcare wearables, specifically those supported by established biomedical signal processing pipelines for feature extraction.

To better reflect this, we have revised the relevant statement in the manuscript to:

"Unlike existing methods that are typically specialized for individual device types/modalities [36, 10], our framework is generalizable across a wide range of healthcare wearables that are supported by well-documented, open-source signal processing pipelines for extracting clinically meaningful features."

To further support this point, we have included an additional experiment using PPG data, expanding beyond the originally reported ECG, EEG, and IMU modalities. Below is the updated Table 2 in the main paper incorporating PPG results.

Table 5: **Comparison results based on self-supervised learning.** To test the efficacy of the downstream knowledge learned during self-supervised learning, we froze the backbone as a feature extractor, and applied KNN and a linear classifier for supervised training. We compared the class-average F1 on the test subset based on the model with the best F1 value on validation subset. Each method was run with five different random seeds to assess the performance. We reported the mean ± standard deviation of the five results. **Bold** indicates the best result, and Underline indicates the second best result.

Methods	KNN				Linear Probing			
	**ECG**	**EEG**	**IMU**	**PPG**	**ECG**	**EEG**	**IMU**	**PPG**
Fully Sup.	-	-	-	-	0.641±0.02	0.658±0.01	0.484±0.05	0.520±0.04
Domain Feat.	0.489±0.05	0.392±0.02	0.470±0.05	0.373±0.04	0.509±0.04	0.548±0.04	0.422±0.06	0.377±0.05
SimCLR [18]	0.427±0.03	0.574±0.02	0.393±0.04	0.395±0.10	0.532±0.07	0.617±0.03	0.433±0.03	0.414±0.05
BYOL [19]	0.380±0.05	0.445±0.09	0.418±0.10	0.243±0.12	0.428±0.04	0.431±0.10	0.449±0.09	0.326±0.08
MoCo [20]	0.450±0.02	0.568±0.04	0.408±0.04	0.368±0.05	0.524±0.05	0.594±0.08	0.425±0.01	0.365±0.04
NNCLR [21]	0.439±0.04	0.511±0.04	0.383±0.06	0.394±0.07	0.517±0.05	0.555±0.03	0.387±0.04	0.355±0.06
TS [22]	0.474±0.05	0.580±0.10	0.449±0.03	0.395±0.04	0.549±0.10	0.627±0.03	0.501±0.06	0.452±0.03
SwAV [13]	0.452±0.10	0.561±0.05	0.438±0.04	0.343±0.02	0.510±0.08	0.638±0.10	0.463±0.07	0.406±0.03
AMCL [14]	0.402±0.06	0.570±0.07	0.413±0.02	0.301±0.05	0.503±0.04	0.626±0.05	0.447±0.05	0.419±0.07
CLOCS [6]	0.488±0.01	0.581±0.06	0.451±0.05	0.376±0.05	0.537±0.03	0.642±0.05	0.483±0.05	0.451±0.06
TFC [12]	0.405±0.03	0.543±0.03	0.267±0.04	0.383±0.04	0.411±0.02	0.571±0.07	0.307±0.01	0.401±0.04
SoftIns [15]	0.481±0.04	0.539±0.03	0.420±0.05	0.337±0.05	0.557±0.10	0.602±0.03	0.498±0.08	0.382±0.06
RNC [25]	0.478±0.04	0.504±0.08	0.465±0.01	0.342±0.02	0.550±0.03	0.560±0.02	0.517±0.05	0.424±0.05
MOMENT [16]	0.443±0.03	0.464±0.02	**0.517**±0.02	0.366±0.01	0.514±0.03	0.456±0.01	0.522±0.02	0.411±0.03
Chronos [17]	0.325±0.03	0.452±0.04	0.445±0.04	0.387±0.05	0.301±0.08	0.406±0.06	0.481±0.06	0.410±0.10
Ours	**0.509**±0.04	**0.591**±0.07	0.465±0.02	**0.420**±0.04	**0.574**±0.03	**0.643**±0.03	**0.526**±0.04	**0.499**±0.02

C 2.2 — The adjective "semantic" is mentioned 33 times. Given its role in the concept formulations and result presentations, a definition is required. For instance (from the introduction) improved search for "local semantic relationship" is claimed as a novel contribution. In the rest of the paper they mention "intricate semantic relationships" as well as "local" and "global" semantic relationships. Last but not least, why "..Data augmentation techniques, including temporal jittering, scaling, magnitude warping, Gaussian noise, and cutout, were applied to generate diverse views of the data following previous works" would not affect the "semantic relationship" of features ?. This is a problematic aspect in the comprehension of the paper and its parts.

Reply: A very good point! We would like to respond to the comment from two aspects.

(1) How to interpret "Semantics" in healthcare wearables.

"Semantics" is a well-established concept in natural language processing (NLP) and computer vision (CV), where it refers to the underlying meaning of data—such as the intent behind a sentence or the object category in an image—that aligns with human understanding. In NLP, semantics distinguishes meaningful content from syntactic form, while in CV, it typically pertains to high-level concepts like object identity, in contrast to low-level visual patterns such as texture or color.

These ideas have been increasingly extended to other domains, including machine learning for healthcare and wearable sensing. For example, Guo *et al.* [44] explored semantic representations in physiological signal analysis, where the goal is to extract clinically relevant information beyond superficial signal characteristics.

In this work, we consider the semantics of wearable waveforms as the clinically meaningful properties encoded in the signals—those that inform medical diagnosis, disease monitoring, or health-related behavior interpretation. In ECG, this might include rhythm regularity and wave morphology, while in EEG it may relate to brainwave patterns associated with sleep stages. They are aligned with how clinical experts interpret these time series to uncover their health-related information. In practice, several commonly used digital biomarkers have been associated with specific waveforms to quantify this semantic information—for instance, standard deviation of RR interval to characterize heart rate variability derived from ECG signals. In other words, these semantically related biomarkers are considered as domain features.

To facilitate readability, we clarify this definition at the beginning of the paper:

"In this context, a key challenge lies in actually capturing the *semantics* of these wearable waveform signals, which is the underlying meaning or relevant health/medical information they convey. More specifically, semantic information refers to high-level abstractions aligned with human understanding and clinical knowledge, such as identifying a specific cardiac arrhythmia from cardiac signals, distinguishing sleep stages from brain signals, or profiling behavioral patterns from movement signals. This is in contrast to *syntactic* features, which describe low-level signal characteristic *syntactic* features (e.g., signal general amplitude or sampling frequency) in the raw waveforms. "

(2) What is the relationship between "Semantic Relationship" and "Data Augmentation".

This is a very insightful point raised concerning.

We agree that potential inappropriate augmentations could potentially distort semantic relationships. Feasible augmentation should mimic wearable signal perturbation, yet not affecting its underlying semantic information, which is also how the self-supervised contrastive learning functions.

In our context, we follow a standard set of augmentations commonly used in prior contrastive learning studies for time series data [6, 12], such as jittering, scaling, and cutout. These augmentations are designed to simulate signal variability without altering the underlying clinical meaning (i.e., they are assumed to be "label-preserving").

One proxy measure of whether the augmentations preserve semantic relationships is to evaluate their impact on downstream classification performance—if the augmentations distort critical features, performance would degrade accordingly.

To address this concern empirically, we have included a new ablation experiment (Table 6, placed in the Supplementary) evaluating the impact of each augmentation, both individually and in combination, on model performance in both supervised and self-supervised settings. These results show that while no single augmentation consistently preserves performance, their combination leads to better feature learning—suggesting that moderate augmentations do not harm semantic alignment in our setting.

Table 6: **Performance of individual and combined augmentations in supervised and self-supervised learning (SimCLR) on CPSC (ECG) dataset.** The performance is reported by class-average F1.

Jittering	Scaling	Warping	Noise	Cutout	Supervised	Self-Supervised
✓					0.561	0.488
	✓				0.558	0.483
		✓			0.557	0.479
			✓		0.553	0.481
				✓	0.556	0.486
✓	✓				0.574	0.497
✓	✓	✓			0.581	0.508
✓	✓	✓	✓		0.588	0.523
✓	✓	✓	✓	✓	0.592	0.532

C 2.3 — Claims that are exaggerated and would require appropriate in-depth analysis of the cited paper. For instance "..deep learning have substantially improved the automatic extraction of semantic information from raw time series data (Ravi, D. et al. 2016)" : the mentioned which is relatively old , does not cover the semantic information of raw signals as processed by DL models. This is a problematic aspect in the comprehension of the paper and its parts.

Reply:

Thank you. In line with our interpretation of "semantics" in C 2.2, we have rephrased the relevant paragraph as below,

Page 1 "In this context, a key challenge lies in actually capturing the *semantics* of these wearable waveform signals, which is, the underlying meaning or clinically relevant information they convey. Semantic information refers to higher-level abstractions aligned with human understanding and clinical knowledge, such as identifying a specific cardiac arrhythmia from cardiac signals, distinguishing sleep stages from brain signals, or profiling behavioral patterns from movement signals. This is in contrast to *syntactic* features, which describe low-level signal characteristic *syntactic* features, which represent low-level patterns or structures in the data (e.g., signal general amplitude or sampling frequency)."

Recent advancements in deep learning have substantially improved the automatic extraction of semantic information from raw time series data, such as ECG [45, 46, 47] and EEG [48, 49], and sometimes even even surpassing human expert performance [46]...

We have also carefully looked into the other parts throughout the paper.

C 2.4 — The word "standard" or "established" are mentioned 4 times, each, in key contexts without expanding what is meant or given an example or a reference. For instance: "..across

all datasets, standard processing was applied." or "Domain-knowledge-informed features were extracted from each modality using established toolboxes and following clinical guidelines." This is a problematic aspect in the comprehension and replicability of the paper experiments. Experimental material and code should include all the necessary information for replicability.

Reply: Thank you for this important comment.

We try to address this concern in the following ways:

(1) Yes, we agree the use of terms like "standard" and "established" could be ambiguous without appropriate context or references. In response, we have carefully revised the manuscript, especially the "Introduction" to explicitly explain this,

"Stepping back from the success of deep learning, conventional supervised learning methodologies for healthcare wearables have heavily relied on manually crafted features based on domain-specific knowledge, as shown in the top panel of Figure 9. Data analysts extract these features with the help of domain expertise, which often refer to "textbook-level" medical knowledge, or involve domain experts such as cardiologists for ECG. These features, spanning morphological, temporal, spectral, signal quality dimensions, etc., are then used in classical machine learning classifiers, such as support vector machines or tree-based methods [50]. More importantly, these features can be readily extracted using well-documented, community-supported open-sourced toolkits, packages, repositories developed specifically for physiological signal analysis. For example, the WFDB Toolbox from PhysioNet[3] provides utilities for ECG signal processing, such as R-peak detection, interval measurement, and signal quality assessment, following clinical best practices, and is widely adopted in the wearable research communities [51]. This ecosystem is further supported by several community-driven initiatives that have developed robust repositories dedicated to domain-specific feature engineering [52, 53, 2, 54]."

(2) To further promote transparency and replicability, we have expanded the Supplementary Materials to include detailed experimental configurations, feature descriptions, and preprocessing pipelines, as shown in the Supplementary Sections S1 and S2. All code including data processing scripts will be made publicly available upon publication.

(3) Additionally, we also provide a curated list of widely-used, community-supported signal processing toolkits for different wearable sensing modalities, which may serve as a useful reference for future work in this area.

C 2.5 — "Domain Expertise Informed Features": the reported features are domain agnostics and they may be relevant for a particular (sub)domain and application only if a meaning is formulated and associated.

Reply: We appreciate this insightful comment.

We acknowledge these features are domain-specific, mostly engineered based on the specific clinical expertise for interpreting these signals. These include well-established biomarkers such as heart rate variability in ECG, and spectral power bands in EEG, which are widely used to interpret signal-level physiological meaning.

A key strength of our framework lies in the observation that this kind of conventional feature engineering has been refined over decades and is now well-supported by open-source toolkits, libraries, and repositories. These features can be easily extracted and have been commonly adopted as clinically meaningful "biomarkers" across various applications.

[3] https://physionet.org/content/wfdb-matlab/0.10.0/

Table 7: Representative open-source toolkits for wearable signal preprocessing and feature extraction, along with supported wearable sensing modalities, including ECG, PPG, EEG, IMU, RSP (Respiratory), ABP (Arterial Blood Pressure), EDA (Electrodermal Activity), MEG (Magnetoencephalography), fNIRS (functional Near-Infrared Spectroscopy).

Toolbox / Package	ECG	PPG	EEG	IMU	RSP	Others
WFDB Toolbox[1]	✓	✓	✓		✓	ABP, EMG
NeuroKit2[2]	✓	✓			✓	EDA , EMG
HeartPy[3]	✓	✓				
BioSPPy[4]	✓	✓	✓		✓	EDA, EMG, PCG
pyHRV[5]	✓	✓				
PyPhysio[6]	✓	✓		✓		EDA, fNIRS
BIOBSS[7]	✓	✓		✓		EDA
MNE-Python[8]			✓			MEG , ECoG, fNIRS
EEGLAB[9]			✓			MEG, ECoG
PhysioKit[10]		✓			✓	EDA
Vital_Sqi[11]	✓	✓				
PPGFeat[12]		✓				

[1] https://physionet.org/content/wfdb-matlab/0.10.0/
[2] https://github.com/neuropsychology/NeuroKit
[3] https://python-heart-rate-analysis-toolkit.readthedocs.io/en/latest/
[4] https://github.com/scientisst/BioSPPy
[5] https://pyhrv.readthedocs.io/en/latest/
[6] https://gitlab.com/a.bizzego/pyphysio
[7] https://github.com/obss/BIOBSS
[8] https://mne.tools/stable/
[9] https://sccn.ucsd.edu/eeglab/
[10] https://github.com/PhysiologicAILab/PhysioKit
[11] https://github.com/meta00/vital_sqi
[12] https://github.com/saadsur/PPGFeat

We fully agree that such domain-informed features may not be sufficiently discriminative for a specific disease classification task—an observation that is also supported by our results in Table ??, where domain features alone underperform compared to full supervision. This naturally raises an important question: **Does our framework require highly discriminative domain features in order to be effective?**

To investigate this, we conducted an analysis presented in Figure 7. We organized the domain features into four groups and assessed their contributions, both individually and in combination, to downstream classification tasks. The results show that even relatively simple or unoptimized feature sets contribute positively when integrated into our self-supervised learning framework. For example, the group with the lowest classification performance still reached an F1 score of 0.545, compared to 0.532 achieved by a conventional SimCLR baseline. This indicates that our framework does not rely heavily on the discriminativeness of domain features.

Below are corresponding discussions in the main paper

Page 11 "**Sensitivity to Domain Features.** Recognizing the critical role of domain features within our integrated framework, we conducted a comprehensive ablation study on these features in the ECG domain. Specifically, we categorized the features into distinct groups (AF related features, Morphological features, RR Interval related features, Beat similarity related features) and systematically ablated each group individually. Our results, shown in Figure 7, illustrate that, traditional logistic regression model trained directly on domain features (in blue) exhibits significant drops in F1 score when any group is removed. This reflects a strong dependency on the presence of specific clinical descriptors. By

contrast, our self-supervised contrastive learning (SSCL) approach (in green) remains robust, consistently outperforming direct regression even with reduced or non-optimized feature sets. For example, the weakest feature group under SSCL still achieved 0.545 in F1 score, exceeding the baseline of 0.532 from SimCLR. This indicates that our framework can effectively leverage domain signals—even when they are coarse or weak—without requiring highly discriminative feature design.

This robustness is clinically meaningful. In real-world wearable settings, data quality often varies, and key features—such as clean RR intervals or stable waveform segments—may be degraded by noise, motion artifacts, or incomplete recording. Moreover, the extracted features, while widely used and clinically grounded, may not be tailored to any specific disease. Our framework's ability to tolerate such imperfections and still extract meaningful representations makes it particularly well-suited for practical deployment in diverse and uncontrolled environments."

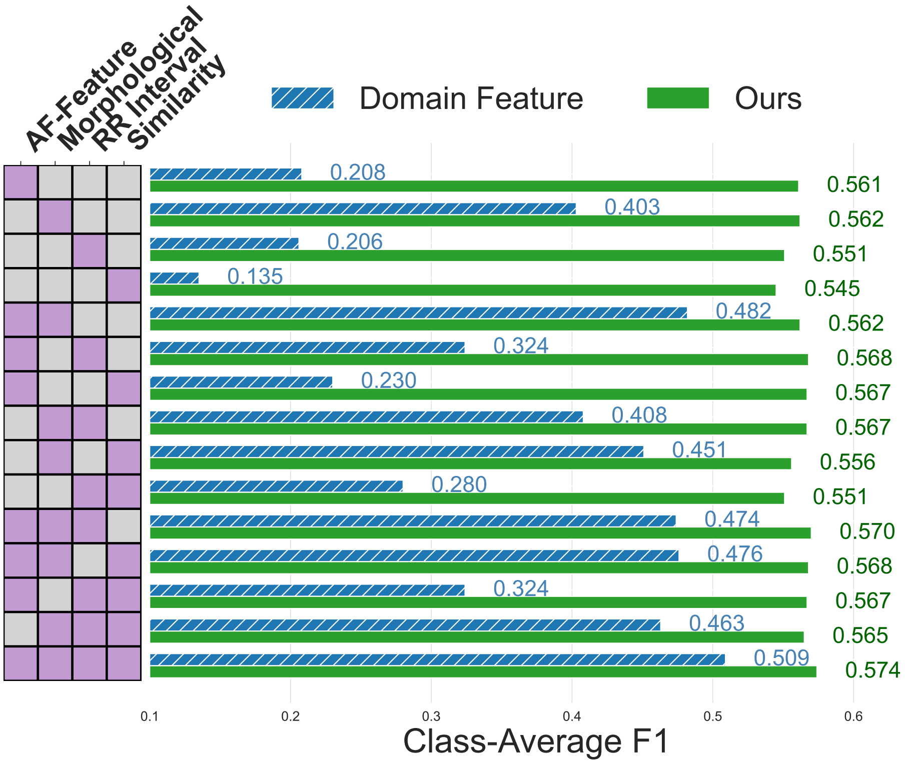

Figure 7: **Comparison of feature group importance for direct logistic regression versus our framework, on CPSC.** The heatmap on the left illustrates which combinations of features (AF-Feature, Morphological, RR Interval, and Similarity) were used, where the purple cubes indicate feature utility. The bar plot on the right shows the class-average F1 score for each feature combination, with direct logistic regression represented in blue (hatched) and our SSCL method in green. Higher values denote better performance, highlighting the advantage of integrating domain features into a self-supervised learning framework for downstream classification tasks.

Having said, we do agree this would be one possible limitation, for those signals that may lack enough ready-to-use resources to extract domain features. We incorporated this in our discussion.

"While this work presents a promising framework, several limitations should be acknowledged. First, the method's reliance on domain-specific features, such as RR intervals in ECG data or spectral power

in EEG, means that its success is closely tied to the quality of these features. In scenarios where domain-specific features are less well-defined or unavailable, the model's performance may suffer."

C 2.6 — Overall Self-Supervised Learning Performance: for fairness comparisons with latest deep representation learning models should be included in the experimental plan. Also authors should comment on the types of models reported in Table 2 and whether those results are replicated from original papers or else.

Reply: Thank you. Indeed, this is a contrustive and fair point! Similar to the response to C 1.8, we have tried to address your concern in the following two aspects.

(1) Expanded comparisons: We have extended our experimental plan to include additional recent deep representation learning models, particularly foundation models designed for time series data (e.g., MOMENT [16] and Chronos [17]). These models are now included in Table 1, allowing for a more comprehensive and fair comparison with the current state-of-the-art in self-supervised learning. We also included comparison of wearable-modality-specific foundation models in Supplementary Material Section S4.1.

In specific, they include:

a) Inclusion of more time-series specific methods: Original implementations include CLOCS[6], originally developed by our group, specifically designed for self-supervised ECG classification, and TFC[12] targets alignment by aligning temporal and frequency level representations for time-series inputs. In addition, we implemented SoftIns from Lee et al. [15], which assigns continuous similarity weights to positive and negative pairs based on their original signal-space distance, characterized using Dynamic Time Warping.

b) Comparison with general-purpose time series foundation model in the main paper:

We have incorporated evaluations of two recent state-of-the-art general-purpose time series foundation models—MOMENT[16] and Chronos[17]—within the main paper. These models are pretrained on large-scale, heterogeneous time series datasets and represent the latest advances in generalizable time series representation learning.

Results regarding a)and b) are presented in Table 5 (Table 2 in the main paper).

c) Comparison with wearable-specific foundation model in the Supplementary: We additionally include results in the Supplementary comparing our method against modality-specific pretrained models designed for waveforms [26, 27]. While such models are often tuned for particular sensor types or clinical tasks and thus may not generalize across modalities, we still evaluated them to provide additional insight.

In detail, we compared with two wearable-modality-specific foundation models (ECGFM [26] for 12-Lead Diagnostic ECG and PaPaGei [27] for PPG). The results, measured in terms of class-average F1 score, are summarized below, with our model pretrained using self-supervised learning. For ECGFM, we used the publicly available pretrained model[4] and adapted our data by duplicating the single-channel ECG signals to 12 channels to match the model's input requirements. For PaPaGei, we followed the official implementation pipeline[5] for PPG representation extraction and evaluation.

As shown in Table 8, ECGFM performs competitively on ECG datasets and occasionally surpasses our method, particularly on CinC17. On the other hand, PaPaGei also shows reasonable performance on PPG signals.

[4]https://github.com/bowang-lab/ECG-FM

[5]https://github.com/Nokia-Bell-Labs/papagei-foundation-model

Please noted that ECGFM was pretrained on the CPSC dataset in a self-supervised manner, and since the exact pretraining setup is not fully disclosed, it is possible that test data may have been included during pretraining. It is also important to note that direct comparisons with these large foundation models are not entirely equitable, due to differences in model size, training scale, and modality-specific design. Our method, by contrast, is lightweight and generalizable across multiple wearable modalities. Incorporating our domain-guided strategies into future foundation model development may enhance both their interpretability and clinical applicability.

Table 8: **Comparison with wearable-modality-specific foundation models. We evaluate our self-supervised pretrained model against ECGFM [26] and PaPaGei [27], two foundation models tailored for 12-lead ECG and PPG signals, respectively.** Performance is reported as class-average F1 score using both KNN and linear probing.

**Method**	**KNN**			**Linear Probing**		
	ECG (CPSC)	ECG (CinC17)	PPG (SimBand)	ECG (CPSC)	ECG (CinC17)	PPG (SimBand)
PaPaGei[27]	-	-	0.335	-	-	0.417
ECGFM[26]	0.511	0.565	-	0.557	0.582	-
Ours	0.509	0.571	0.420	0.574	0.540	0.499

(2) Clarified implementation details: For all compared methods reported in Table 1, we explicitly clarify in the Methods and Supplementary Materials whether results were reproduced by us or directly reported from original papers. The majority of experiments, including those for SimCLR, BYOL, MoCo, CLOCS, and SoftIns, were reproduced using publicly available code and re-implemented under a unified pipeline using the same backbone (ResNet1d18), preprocessing steps, and evaluation criteria to ensure fairness.

We will provide the preprocessing script and feature extraction code for benchmarking upon publication.

C 2.7 — t-SNE plots in Figure 4 are not useful for contrasting the two approaches.

Reply: Thanks for pointing this out. Yes, we have refined the figure (also refer to C 1.16 and C 1.17), to better illustrate the advantages of our method, based on Capture24.

Page 7 "In detail, the revised figure represents a t-SNE visualization (Figure 8) comparing domain features, features from end-to-end supervised learning, those learned by SimCLR, and those learned by our method, based on the whole dataset of Capture24. It can be observed from the first column, that domain knowledge based features are evenly distributed between training/validation/testing subjects. In contrast, features learned via end-to-end supervised training (second column) exhibit clear clustering based on training and validation/test splits. This suggests that the model may have overfitted to subject-specific characteristics, such as individual wearing patterns or sensor noise, which are not directly related to activity related semantic information."

"As shown in the third and fourth column, SimCLR and Ours, both approaches produce more evenly distributed feature representations across training, validation, and test sets. SimCLR performs instance-level contrast, which implicitly push away the representations of the instances from the same subject. This helps to avoid learning those subject-specific characteristics as biases. However, the learned representation are not semantically discriminative enough. As observed from the plot of second row, third column, points from different classes get more mixed compared to other plots of the same

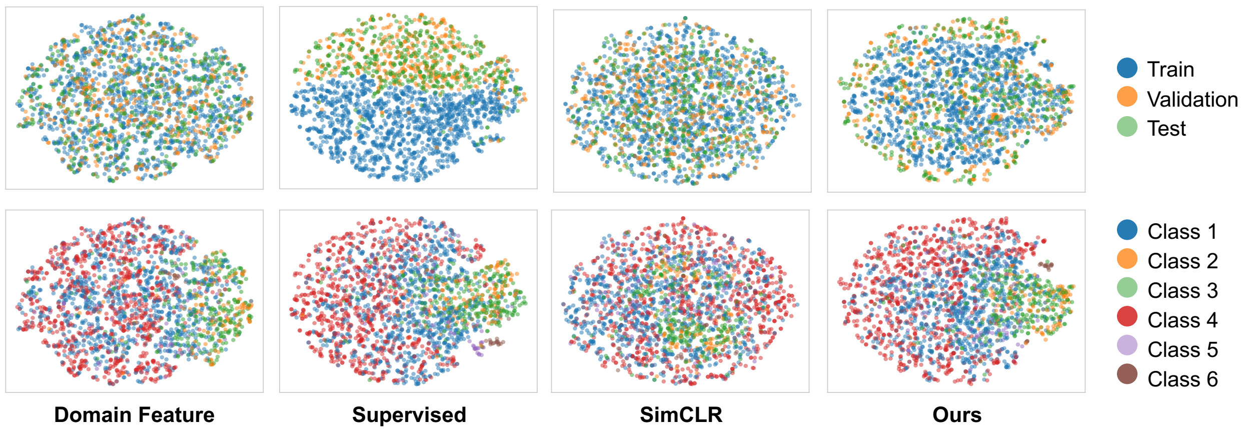

Figure 8: **t-SNE visualizations of domain features, features extracted from end-to-end supervised learning, from SimCLR and from our method, respectively.** Each point corresponds to a sample from the Capture24 dataset. Each column represents the same plots, with points colorized by different manners. In the top panel, each sample points were colored by data split (train/validation/test), whilst in the bottom, shaped by class label.

row. Our method goes a step further, by using domain features to guide positive pair selection. This helps the model focus on learning semantically meaningful patterns rather than unrelated differences between individuals."

C 2.8 — Fig.1: Graphics are not clear. Specifically in the c) part, middle part and rightmost part the relation of color, edges, features is messy.

Reply: Thank you very much for pointing this out! In line with Reviewer 1 C 1.3, we have revised the original Figure 1 to two subfigures, as shown in Figures 9 and 10.

In particular, the updated Figure 9 illustrates the difference between traditional ("old-school") manual feature engineering and modern ("new-school") self-supervised contrastive learning (SSCL), and why the implicit domain expertise encoded in domain features is important, in terms of the shortcomings of "new-school" self-supervised contrastive learning. In particular, the figure emphasizes the issue that SSCL typically treats all non-identical samples as negatives, even when they may share important clinical similarities.

In addition, Figure 10 more clearly illustrates the four key components of our proposed framework: (a) offline extraction and clustering of domain-informed features, (b) augmentation and shared encoding of input signals, (c) instance-level contrast guided by nearest neighbours in the domain feature space, and (d) prototype-level contrast based on domain-guided clustering. We believe this clearer breakdown will help readers better understand how domain knowledge is integrated throughout the self-supervised learning process.

C 2.9 — "we conducted experiments across three types of wearable sensors: electrocardiogram (ECG), electroencephalogram (EEG), and inertial measurement unit (IMU)." . .ECG etc. are signals and not sensors.

Reply: Thanks, this has been rectified.

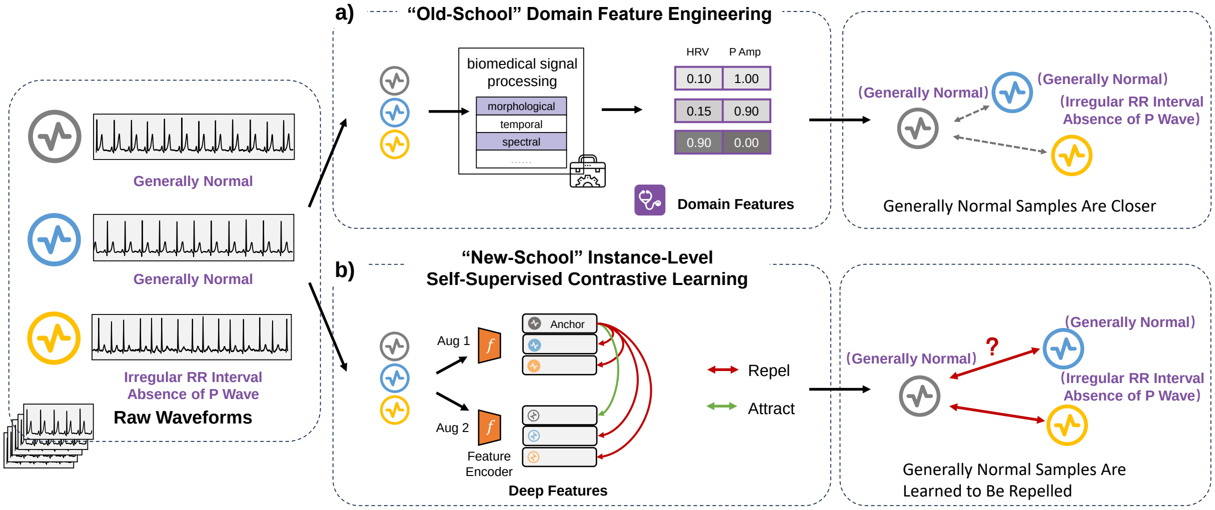

Figure 9: **Illustration of the pipelines of "old-school" domain feature engineering and "new-school" self-supervised contrastive learning (SSCL), for healthcare wearables.** Take electrocardiogram (ECG) as an example, a common wearable modality for detecting cardiovascular abnormalities. Notably, both pipelines function without necessarily requiring access to ground-truth labels. **a)** In the "old-school" pipeline, domain features such as heart rate variability (HRV), and the presence or amplitude of P-waves can be extracted using off-the-shelf biomedical signal processing toolboxes. These features are grounded in clinical knowledge and have long been used to support cardiovascular diagnosis. For instance, a normal reference ECG with stable RR intervals and clear P-waves would be grouped closer to another normal ECG with similar physiological characteristics, and distinctly separated from a abnormal ECG showing irregular rhythms or missing waveform components. **b)** In the "new-school" SSCL pipeline, representations are learned by contrasting augmented views of unlabeled data. Specifically, for each input signal, two augmented views (Aug 1 and Aug 2) are generated and passed through a shared encoder. The model is trained using a contrastive loss to pull the positive pair (the two augmentations) closer in representation space, while pushing them away from all other instances in the batch (treated as negative pairs), regardless of their physiological similarity. This could yield suboptimal representations. For example, a normal anchor ECG may be treated as equally dissimilar to both a generally normal sample and a clinically abnormal one, despite their differing clinical significance.

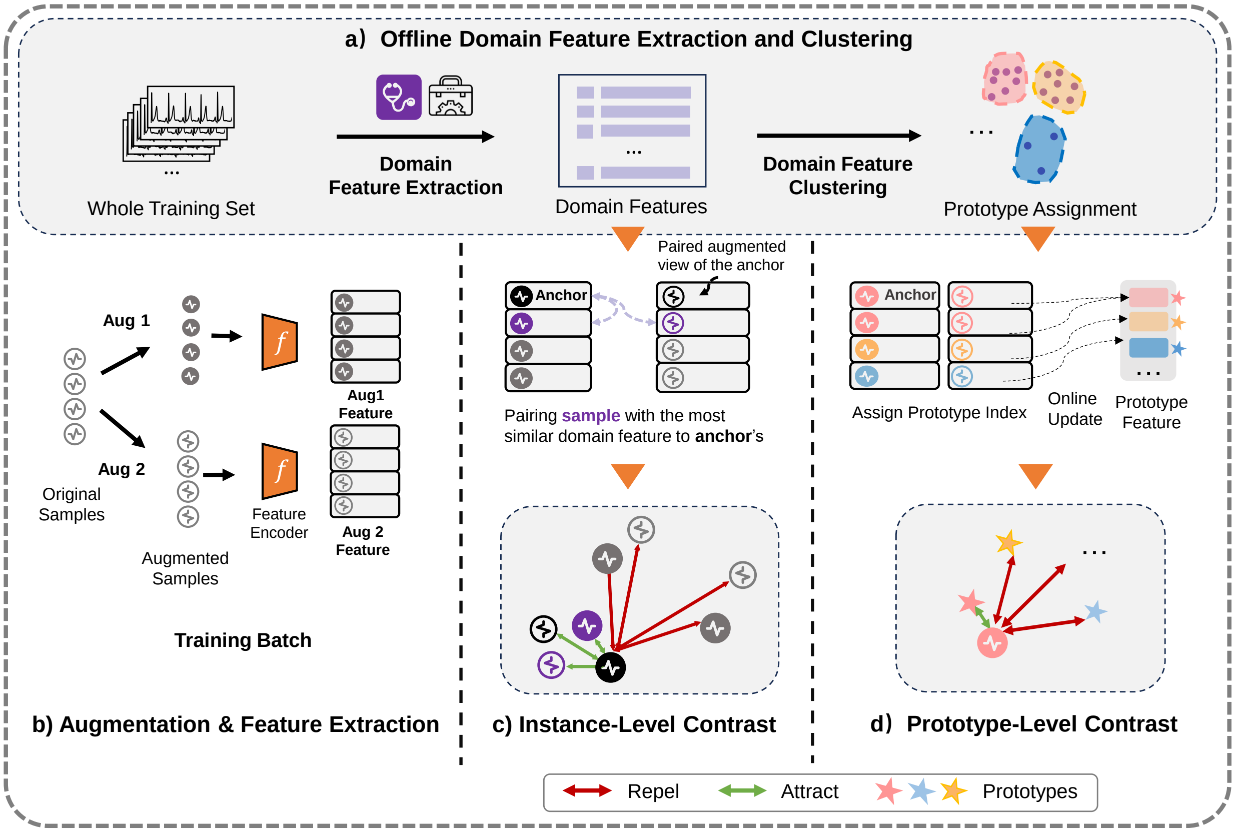

Figure 10: **Illustration of our domain-knowledge-guided self-supervised contrastive learning framework for decoding healthcare wearable time series data.** It incorporates medical domain expertise, which is implicitly "embedded" in "old-school" domain feature engineering, into a "new-school" self-supervised contrastive learning (SSCL) framework for decoding wearable waveforms. **a) Offline Domain Feature Extraction and Clustering.** Before training, we extract domain knowledge-informed bespoke features from all input training samples following domain-specific biomedical signal processing practices and toolboxes. These features are subsequently clustered via offline k-means to assign each sample to a domain-feature based prototype group. **b) Augmentation and Deep Feature Extraction.** During training, within each batch, each input signal is augmented twice and passed through a shared encoder to produce latent representations, following a standard instance-level self-supervised contrastive learning pipeline. **c) Instance-Level Contrast.** In addition to treating paired augmented views as a positive pair (marked in black), the nearest neighbour in the domain feature space is selected and its two augmented views (marked in purple) are included as additional positive. This enhances physiological consistency by preventing clinically similar samples from being treated as negatives. **d) Prototype-Level Contrast.** Subsequently, based on the preassigned prototype index from offline clustering, each prototype representation is updated online using an exponential moving average of the encoded features from its assigned samples. These prototypes capture and maintain the global structure of the training data. During training, each sample is encouraged to align with its corresponding domain-informed prototype, promoting consistency across the feature space.

References

[1] J. Lai, H. Tan, J. Wang, L. Ji, J. Guo, B. Han, Y. Shi, Q. Feng, and W. Yang, "Practical intelligent diagnostic algorithm for wearable 12-lead ecg via self-supervised learning on large-scale dataset," *Nature Communications*, vol. 14, no. 1, p. 3741, 2023.

[2] S. Chan, H. Yuan, C. Tong, A. Acquah, A. Schonfeldt, J. Gershuny, and A. Doherty, "Capture-24: A large dataset of wrist-worn activity tracker data collected in the wild for human activity recognition," 2024.

[3] X. Gu, F. Deligianni, J. Han, X. Liu, W. Chen, G.-Z. Yang, and B. Lo, "Beyond supervised learning for pervasive healthcare," *IEEE Reviews in Biomedical Engineering*, 2023.

[4] B. Kemp, A. H. Zwinderman, B. Tuk, H. A. Kamphuisen, and J. J. Oberye, "Analysis of a sleep-dependent neuronal feedback loop: the slow-wave microcontinuity of the eeg," *IEEE Transactions on Biomedical Engineering*, vol. 47, no. 9, pp. 1185–1194, 2000.

[5] S. Abbaspourazad, O. Elachqar, A. Miller, S. Emrani, U. Nallasamy, and I. Shapiro, "Large-scale training of foundation models for wearable biosignals," in *The Twelfth International Conference on Learning Representations*, 2024. [Online]. Available: https://openreview.net/forum?id=pC3WJHf51j

[6] D. Kiyasseh, T. Zhu, and D. A. Clifton, "CLOCS: contrastive learning of cardiac signals across space, time, and patients," in *Proceedings of the 38th International Conference on Machine Learning, ICML 2021, 18-24 July 2021, Virtual Event*, ser. Proceedings of Machine Learning Research, M. Meila and T. Zhang, Eds., vol. 139. PMLR, 2021, pp. 5606–5615. [Online]. Available: http://proceedings.mlr.press/v139/kiyasseh21a.html

[7] X. Lan, D. Ng, S. Hong, and M. Feng, "Intra-inter subject self-supervised learning for multivariate cardiac signals," in *Thirty-Sixth AAAI Conference on Artificial Intelligence, AAAI 2022, Thirty-Fourth Conference on Innovative Applications of Artificial Intelligence, IAAI 2022, The Twelveth Symposium on Educational Advances in Artificial Intelligence, EAAI 2022 Virtual Event, February 22 - March 1, 2022.* AAAI Press, 2022, pp. 4532–4540. [Online]. Available: https://ojs.aaai.org/index.php/AAAI/article/view/20376

[8] T. Huynh, S. Kornblith, M. R. Walter, M. Maire, and M. Khademi, "Boosting contrastive self-supervised learning with false negative cancellation," in *Proceedings of the IEEE/CVF winter conference on applications of computer vision*, 2022, pp. 2785–2795.

[9] J. Han, X. Gu, and B. Lo, "Semi-supervised contrastive learning for generalizable motor imagery eeg classification," in *2021 IEEE 17th International Conference on Wearable and Implantable Body Sensor Networks (BSN).* IEEE, 2021, pp. 1–4.

[10] B. Gopal, R. Han, G. Raghupathi, A. Ng, G. Tison, and P. Rajpurkar, "3kg: Contrastive learning of 12-lead electrocardiograms using physiologically-inspired augmentations," in *Machine learning for health.* PMLR, 2021, pp. 156–167.

[11] C. Zhao, W. Wu, H. Zhang, R. Zhang, X. Zheng, and X. Kong, "Sleep stage classification via multi-view based self-supervised contrastive learning of eeg," *IEEE Journal of Biomedical and Health Informatics*, 2024.

[12] X. Zhang, Z. Zhao, T. Tsiligkaridis, and M. Zitnik, "Self-supervised contrastive pre-training for time series via time-frequency consistency," in *Advances in Neural Information Processing Systems 35: Annual Conference on Neural Information Processing Systems 2022, NeurIPS 2022, New Orleans, LA, USA, November 28 - December 9, 2022*, S. Koyejo, S. Mohamed, A. Agarwal, D. Belgrave, K. Cho, and A. Oh, Eds., 2022. [Online]. Available: http://papers.nips.cc/paper_files/paper/2022/hash/194b8dac525581c346e30a2cebe9a369-Abstract-Conference.html

[13] M. Caron, I. Misra, J. Mairal, P. Goyal, P. Bojanowski, and A. Joulin, "Unsupervised learning of visual features by contrasting cluster assignments," in *Advances in Neural Information Processing Systems 33: Annual Conference on Neural Information Processing Systems 2020, NeurIPS 2020, December 6-12, 2020, virtual*, H. Larochelle, M. Ranzato, R. Hadsell, M. Balcan, and H. Lin, Eds., 2020. [Online]. Available: https://proceedings.neurips.cc/paper/2020/hash/70feb62b69f16e0238f741fab228fec2-Abstract.html

[14] L. Wang, P. Koniusz, T. Gedeon, and L. Zheng, "Adaptive multi-head contrastive learning," in *European Conference on Computer Vision.* Springer, 2024, pp. 404–421.

[15] S. Lee, T. Park, and K. Lee, "Soft contrastive learning for time series," in *The Twelfth International Conference on Learning Representations*, 2024. [Online]. Available: https://openreview.net/forum?id=pAsQSWlDUf

[16] M. Goswami, K. Szafer, A. Choudhry, Y. Cai, S. Li, and A. Dubrawski, "Moment: a family of open time-series foundation models," in *Proceedings of the 41st International Conference on Machine Learning*, 2024, pp. 16 115–16 152.

[17] A. F. Ansari, L. Stella, A. C. Turkmen, X. Zhang, P. Mercado, H. Shen, O. Shchur, S. S. Rangapuram, S. P. Arango, S. Kapoor, J. Zschiegner, D. C. Maddix, H. Wang, M. W. Mahoney, K. Torkkola, A. G. Wilson, M. Bohlke-Schneider, and B. Wang, "Chronos: Learning the language of time series," *Transactions on Machine Learning Research*, 2024, expert Certification. [Online]. Available: https://openreview.net/forum?id=gerNCVqqtR

[18] T. Chen, S. Kornblith, M. Norouzi, and G. E. Hinton, "A simple framework for contrastive learning of visual representations," in *Proceedings of the 37th International Conference on Machine Learning, ICML 2020, 13-18 July 2020, Virtual Event*, ser. Proceedings of Machine Learning Research, vol. 119. PMLR, 2020, pp. 1597–1607. [Online]. Available: http://proceedings.mlr.press/v119/chen20j.html

[19] J. Grill, F. Strub, F. Altché, C. Tallec, P. H. Richemond, E. Buchatskaya, C. Doersch, B. Á. Pires, Z. Guo, M. G. Azar, B. Piot, K. Kavukcuoglu, R. Munos, and M. Valko, "Bootstrap your own latent - A new approach to self-supervised learning," in *Advances in Neural Information Processing Systems 33: Annual Conference on Neural Information Processing Systems 2020, NeurIPS 2020, December 6-12, 2020, virtual*, H. Larochelle, M. Ranzato, R. Hadsell, M. Balcan, and H. Lin, Eds., 2020. [Online]. Available: https://proceedings.neurips.cc/paper/2020/hash/f3ada80d5c4ee70142b17b8192b2958e-Abstract.html

[20] K. He, H. Fan, Y. Wu, S. Xie, and R. B. Girshick, "Momentum contrast for unsupervised visual representation learning," in *2020 IEEE/CVF Conference on Computer Vision and Pattern Recognition, CVPR 2020, Seattle, WA, USA, June 13-19, 2020.* IEEE, 2020, pp. 9726–9735. [Online]. Available: https://doi.org/10.1109/CVPR42600.2020.00975

[21] D. Dwibedi, Y. Aytar, J. Tompson, P. Sermanet, and A. Zisserman, “With a little help from my friends: Nearest-neighbor contrastive learning of visual representations,” in *2021 IEEE/CVF International Conference on Computer Vision, ICCV 2021, Montreal, QC, Canada, October 10-17, 2021.* IEEE, 2021, pp. 9568–9577. [Online]. Available: https://doi.org/10.1109/ICCV48922.2021.00945

[22] A. Kukleva, M. Böhle, B. Schiele, H. Kuehne, and C. Rupprecht, “Temperature schedules for self-supervised contrastive methods on long-tail data,” in *The Eleventh International Conference on Learning Representations, ICLR 2023, Kigali, Rwanda, May 1-5, 2023.* OpenReview.net, 2023. [Online]. Available: https://openreview.net/pdf?id=ejHUr4nfHhD

[23] K. He, X. Zhang, S. Ren, and J. Sun, “Deep residual learning for image recognition,” in *Proceedings of the IEEE conference on computer vision and pattern recognition*, 2016, pp. 770–778.

[24] Y. Liang, H. Wen, Y. Nie, Y. Jiang, M. Jin, D. Song, S. Pan, and Q. Wen, “Foundation models for time series analysis: A tutorial and survey,” in *Proceedings of the 30th ACM SIGKDD conference on knowledge discovery and data mining*, 2024, pp. 6555–6565.

[25] K. Zha, P. Cao, J. Son, Y. Yang, and D. Katabi, “Rank-n-contrast: Learning continuous representations for regression,” in *Advances in Neural Information Processing Systems 36: Annual Conference on Neural Information Processing Systems 2023, NeurIPS 2023, New Orleans, LA, USA, December 10 - 16, 2023*, A. Oh, T. Naumann, A. Globerson, K. Saenko, M. Hardt, and S. Levine, Eds., 2023. [Online]. Available: http://papers.nips.cc/paper_files/paper/2023/hash/39e9c5913c970e3e49c2df629daff636-Abstract-Conference.html

[26] K. McKeen, L. Oliva, S. Masood, A. Toma, B. Rubin, and B. Wang, “Ecg-fm: An open electrocardiogram foundation model,” *arXiv preprint arXiv:2408.05178*, 2024.

[27] A. Pillai, D. Spathis, F. Kawsar, and M. Malekzadeh, “Papagei: Open foundation models for optical physiological signals,” in *The Thirteenth International Conference on Learning Representations*, 2025. [Online]. Available: https://openreview.net/forum?id=kYwTmlq6Vn

[28] G. D. Clifford, C. Liu, B. Moody, H. L. Li-wei, I. Silva, Q. Li, A. Johnson, and R. G. Mark, “Af classification from a short single lead ecg recording: The physionet/computing in cardiology challenge 2017,” in *2017 Computing in Cardiology (CinC).* IEEE, 2017, pp. 1–4.

[29] F. Liu, C. Liu, L. Zhao, X. Zhang, X. Wu, X. Xu, Y. Liu, C. Ma, S. Wei, Z. He *et al.*, “An open access database for evaluating the algorithms of electrocardiogram rhythm and morphology abnormality detection,” *Journal of Medical Imaging and Health Informatics*, vol. 8, no. 7, pp. 1368–1373, 2018.

[30] A. E. Johnson, T. J. Pollard, L. Shen, L.-w. H. Lehman, M. Feng, M. Ghassemi, B. Moody, P. Szolovits, L. Anthony Celi, and R. G. Mark, “Mimic-iii, a freely accessible critical care database,” *Scientific data*, vol. 3, no. 1, pp. 1–9, 2016.

[31] S. K. Bashar, D. Han, S. Hajeb-Mohammadalipour, E. Ding, C. Whitcomb, D. D. McManus, and K. H. Chon, “Atrial fibrillation detection from wrist photoplethysmography signals using smartwatches,” *Scientific reports*, vol. 9, no. 1, p. 15054, 2019.

[32] D. Han, S. K. Bashar, F. Mohagheghian, E. Ding, C. Whitcomb, D. D. McManus, and K. H. Chon, "Premature atrial and ventricular contraction detection using photoplethysmographic data from a smartwatch," *Sensors*, vol. 20, no. 19, p. 5683, 2020.

[33] V. Sangha, A. Khunte, G. Holste, B. J. Mortazavi, Z. Wang, E. K. Oikonomou, and R. Khera, "Biometric contrastive learning for data-efficient deep learning from electrocardiographic images," *Journal of the American Medical Informatics Association*, vol. 31, no. 4, pp. 855–865, 2024.

[34] H. Yu, P. Guo, and A. Sano, "ECG semantic integrator (ESI): A foundation ECG model pretrained with LLM-enhanced cardiological text," *Transactions on Machine Learning Research*, 2024. [Online]. Available: https://openreview.net/forum?id=giEbq8Khcf

[35] A. Das, W. Kong, R. Sen, and Y. Zhou, "A decoder-only foundation model for time-series forecasting," in *Forty-first International Conference on Machine Learning*, 2024.

[36] H. Yuan, S. Chan, A. P. Creagh, C. Tong, A. Acquah, D. A. Clifton, and A. Doherty, "Self-supervised learning for human activity recognition using 700,000 person-days of wearable data," *NPJ digital medicine*, vol. 7, no. 1, p. 91, 2024.

[37] R. Zhou, L. Lu, Z. Liu, T. Xiang, Z. Liang, D. A. Clifton, Y. Dong, and Y.-T. Zhang, "Semi-supervised learning for multi-label cardiovascular diseases prediction: a multi-dataset study," *IEEE Transactions on Pattern Analysis and Machine Intelligence*, vol. 46, no. 5, pp. 3305–3320, 2023.

[38] K. Zhang, Q. Wen, C. Zhang, R. Cai, M. Jin, Y. Liu, J. Zhang, Y. Liang, G. Pang, D. Song *et al.*, "Self-supervised learning for time series analysis: Taxonomy, progress, and prospects," *ArXiv preprint*, vol. abs/2306.10125, 2023. [Online]. Available: https://arxiv.org/abs/2306.10125

[39] K. Sohn, D. Berthelot, N. Carlini, Z. Zhang, H. Zhang, C. A. Raffel, E. D. Cubuk, A. Kurakin, and C.-L. Li, "Fixmatch: Simplifying semi-supervised learning with consistency and confidence," *Advances in neural information processing systems*, vol. 33, pp. 596–608, 2020.

[40] Z. Xiao, H. Tong, R. Qu, H. Xing, S. Luo, Z. Zhu, F. Song, and L. Feng, "Capmatch: Semi-supervised contrastive transformer capsule with feature-based knowledge distillation for human activity recognition," *IEEE transactions on neural networks and learning systems*, 2023.

[41] J. Li, P. Zhou, C. Xiong, and S. C. H. Hoi, "Prototypical contrastive learning of unsupervised representations," in *9th International Conference on Learning Representations, ICLR 2021, Virtual Event, Austria, May 3-7, 2021.* OpenReview.net, 2021. [Online]. Available: https://openreview.net/forum?id=KmykpuSrjcq

[42] J. Ren, C. Yu, S. Sheng, X. Ma, H. Zhao, S. Yi, and H. Li, "Balanced meta-softmax for long-tailed visual recognition," in *Advances in Neural Information Processing Systems 33: Annual Conference on Neural Information Processing Systems 2020, NeurIPS 2020, December 6-12, 2020, virtual*, H. Larochelle, M. Ranzato, R. Hadsell, M. Balcan, and H. Lin, Eds., 2020. [Online]. Available: https://proceedings.neurips.cc/paper/2020/hash/2ba61cc3a8f44143e1f2f13b2b729ab3-Abstract.html

[43] M. N. Mohsenvand, M. R. Izadi, and P. Maes, "Contrastive representation learning for electroencephalogram classification," in *Machine Learning for Health*. PMLR, 2020, pp. 238–253.

[44] Z. Guo, C. Ding, X. Hu, and C. Rudin, "A supervised machine learning semantic segmentation approach for detecting artifacts in plethysmography signals from wearables," *Physiological Measurement*, vol. 42, no. 12, p. 125003, 2021.

[45] A. Y. Hannun, P. Rajpurkar, M. Haghpanahi, G. H. Tison, C. Bourn, M. P. Turakhia, and A. Y. Ng, "Cardiologist-level arrhythmia detection and classification in ambulatory electrocardiograms using a deep neural network," *Nature medicine*, vol. 25, no. 1, pp. 65–69, 2019.

[46] A. H. Ribeiro, M. H. Ribeiro, G. M. Paixão, D. M. Oliveira, P. R. Gomes, J. A. Canazart, M. P. Ferreira, C. R. Andersson, P. W. Macfarlane, W. Meira Jr *et al.*, "Automatic diagnosis of the 12-lead ecg using a deep neural network," *Nature communications*, vol. 11, no. 1, p. 1760, 2020.

[47] L. Lu, T. Zhu, A. H. Ribeiro, L. Clifton, E. Zhao, J. Zhou, A. L. P. Ribeiro, Y.-T. Zhang, and D. A. Clifton, "Decoding 2.3 million ecgs: interpretable deep learning for advancing cardiovascular diagnosis and mortality risk stratification," *European Heart Journal-Digital Health*, vol. 5, no. 3, pp. 247–259, 2024.

[48] J. Levy, D. Álvarez, F. Del Campo, and J. A. Behar, "Deep learning for obstructive sleep apnea diagnosis based on single channel oximetry," *Nature Communications*, vol. 14, no. 1, p. 4881, 2023.

[49] H. Phan, O. Y. Chén, M. C. Tran, P. Koch, A. Mertins, and M. De Vos, "Xsleepnet: Multi-view sequential model for automatic sleep staging," *IEEE Transactions on Pattern Analysis and Machine Intelligence*, vol. 44, no. 9, pp. 5903–5915, 2021.

[50] Z. Mei, X. Gu, H. Chen, and W. Chen, "Automatic atrial fibrillation detection based on heart rate variability and spectral features," *IEEE Access*, vol. 6, pp. 53 566–53 575, 2018.

[51] D. Makowski, T. Pham, Z. J. Lau, J. C. Brammer, F. Lespinasse, H. Pham, C. Schölzel, and S. A. Chen, "Neurokit2: A python toolbox for neurophysiological signal processing," *Behavior research methods*, pp. 1–8, 2021.

[52] M. Shao, G. Bin, S. Wu, G. Bin, J. Huang, and Z. Zhou, "Detection of atrial fibrillation from ecg recordings using decision tree ensemble with multi-level features," *Physiological measurement*, vol. 39, no. 9, p. 094008, 2018.

[53] J. Van Der Donckt, J. Van Der Donckt, E. Deprost, N. Vandenbussche, M. Rademaker, G. Vandewiele, and S. Van Hoecke, "Do not sleep on traditional machine learning: Simple and interpretable techniques are competitive to deep learning for sleep scoring," *Biomedical Signal Processing and Control*, vol. 81, p. 104429, 2023.

[54] M. Á. Goda, P. H. Charlton, and J. A. Behar, "pyppg: a python toolbox for comprehensive photoplethysmography signal analysis," *Physiological Measurement*, vol. 45, no. 4, p. 045001, 2024.

Response to review comments

COMMSENG-24-0634A

We thank the reviewer for the thorough reading of our manuscript and providing constructive feedback to further improve our work. Please see our response to the one remaining concern.

Reviewer 1

C 1.1 — I thank the authors for their thorough response. I have carefully reviewed their replies to my concerns and find them convincing; they significantly improve the quality of the submission.

One remaining concern: Figure 2 has improved, but there are still some unclear elements. In the lower part of the figure, different features appear with either filled or unfilled backgrounds and in various colors (purple, black, gray). The meaning of these visual distinctions is not clear— a legend is needed. Additionally, there is an arrow running from top to bottom in the center, but it is not evident how the top and bottom sections are connected, either through color coding or any other means. The connection on the right side is clearer in comparison.

Reply: Many thanks. We have revised the figure by expanding the illustrative legends and clarifying the central connection. Please see the updated figure below.

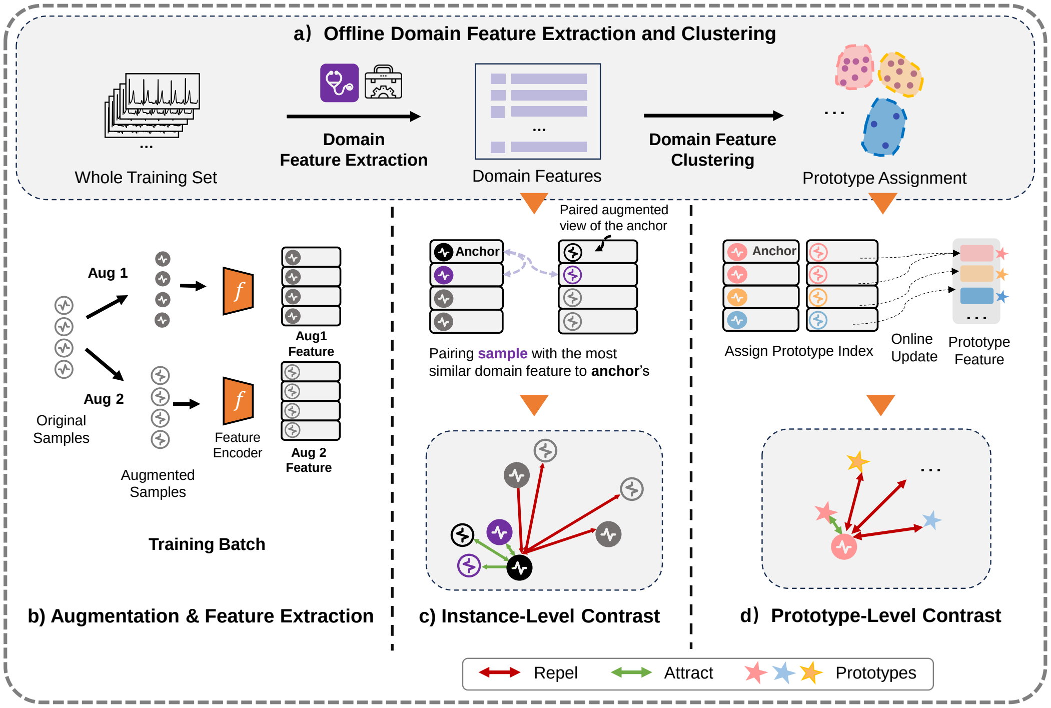

Original Figure 2.

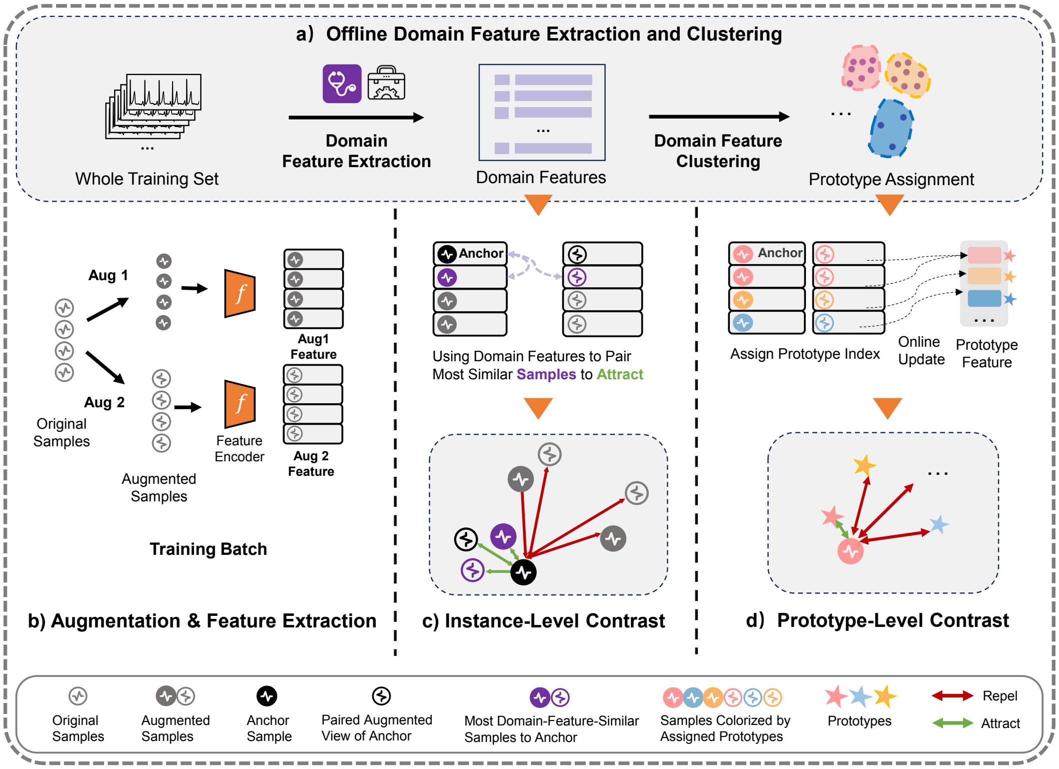

Revised Figure 2.
